# Conditional deletion of miR-204 and miR-211 in murine retinal pigment epithelium results in retinal degeneration

**DOI:** 10.1016/j.jbc.2024.107344

**Published:** 2024-05-04

**Authors:** Samuel W. Du, Ravikiran Komirisetty, Dominik Lewandowski, Elliot H. Choi, Damian Panas, Susie Suh, Marcin Tabaka, Roxana A. Radu, Krzysztof Palczewski

**Affiliations:** 1 Gavin Herbert Eye Institute-Center for Translational Vision Research, Department of Ophthalmology, University of California, Irvine, California, USA; 2 Department of Physiology and Biophysics, University of California, Irvine, California, USA; 3 Department of Ophthalmology and UCLA Stein Eye Institute, David Geffen School of Medicine, University of California, Los Angeles, Los Angeles, California, USA; 4 International Centre for Translational Eye Research, Warsaw, Poland; 5 Institute of Physical Chemistry, Polish Academy of Sciences, Warsaw, Poland; 6 Department of Chemistry, University of California, Irvine, Irvine, California, USA; 7 Department of Molecular Biology and Biochemistry, University of California, Irvine, Irvine, California, USA

**Keywords:** microRNA (miRNA), retinal degeneration, gene KO, RPE, electrophysiology, inflammation

## Abstract

MicroRNAs (miRs) are short, evolutionarily conserved noncoding RNAs that canonically downregulate expression of target genes. The miR family composed of miR-204 and miR-211 is among the most highly expressed miRs in the retinal pigment epithelium (RPE) in both mouse and human and also retains high sequence identity. To assess the role of this miR family in the developed mouse eye, we generated two floxed conditional KO mouse lines crossed to the RPE65-ERT2-Cre driver mouse line to perform an RPE-specific conditional KO of this miR family in adult mice. After Cre-mediated deletion, we observed retinal structural changes by optical coherence tomography; dysfunction and loss of photoreceptors by retinal imaging; and retinal inflammation marked by subretinal infiltration of immune cells by imaging and immunostaining. Single-cell RNA sequencing of diseased RPE and retinas showed potential miR-regulated target genes, as well as changes in noncoding RNAs in the RPE, rod photoreceptors, and Müller glia. This work thus highlights the role of miR-204 and miR-211 in maintaining RPE function and how the loss of miRs in the RPE exerts effects on the neural retina, leading to inflammation and retinal degeneration.

The vertebrate retina is composed of sensory neural circuits and supportive cells, which integrate incoming light from the environment to enable visual and nonvisual behavior. The sensory neurons which drive visual behavior are the rod and cone photoreceptors. Through the isomerization of 11-*cis*-retinal to all-*trans*-retinal, these cells convert photons of light into graded stimuli that are further processed by downstream neurons. The complex function of these highly specialized sensory neurons is dependent on the health and function of the retinal pigment epithelium (RPE), a postmitotic epithelial monolayer derived from the neuroectoderm, which sits directly adjacent to the outer segments of the rod and cone photoreceptors (1). The RPE and photoreceptors are deeply intertwined, both physically and functionally. While the RPE is not known to contribute directly to image-forming vision, its metabolic and signaling pathways directly impact the physiology and function of photoreceptors. These pathways include the visual cycle which renews the supply of 11-*cis*-retinal, transport systems for critical nutrients such as glucose from the circulation, and the phagocytotic system for daily circadian turnover of spent photoreceptor outer segments (2). Continuous crosstalk between the RPE and photoreceptors is necessary for these functions, meaning that dysfunction or death of the RPE leads to subsequent photoreceptor death.

A growing body of evidence has implicated microRNAs (miRs) in the posttranscriptional regulation of gene expression from mRNAs (3, 4, 5). Mature miRs are short noncoding RNAs, usually between 20 and 22 nucleotides long. After transcription by RNA polymerase II, the nascent miRs are processed and cleaved by Drosha/DGCR8 and Dicer into mature miRs. Immature miRs are processed into two mature miRs, the forward position 5p species and the reverse position 3p species, which often contain different seed sequences (6, 7, 8). They subsequently form an RNA-induced silencing complex with argonaute proteins, most prominently AGO2 (9). MiRs classically bind to an 8-nucleotide seed sequence in the 3′-UTR of the target mRNA, promoting its degradation (10). It is thought that miRs provide an additional layer of posttranscriptional control by fine-tuning gene expression, exemplified by the critical role of miR *lin-4* in *Caenorhabditis elegans* development by controlling proliferation and differentiation of specialized tissues in the worm (11, 12). In addition, there are a limited number of reports suggesting alternative roles for miRs in gene expression, including increased translation and upregulation of targeted transcripts (13, 14).

In the eye, miRs play an important role in both development and normal physiology (15). A seminal study demonstrated that the miR processing enzymes Dicer1 and DGCR8 are critical for the maintenance of RPE and retinal health (16, 17). Mice lacking either of these enzymes showed thinning of the outer nuclear layer (ONL), where the photoreceptor nuclei reside, and disruption of the RPE monolayer organization. However, other reports suggest that Dicer1 instead controls *Alu* or *Alu*-like repeat RNA (18). Nevertheless, it has been established that miRs directly influence retinal health; for example, the miR family 182/96/183 is highly expressed in photoreceptors, and the absence of these miRs results in photoreceptor dysfunction (19).

The miR family comprising miR-204 and miR-211 is among the most highly expressed miRs in mouse and human RPE (20, 21). In mice, these two miRs that share an identical seed sequence and their mature sequences differ by only one nucleotide, suggesting they may regulate the same genes. When compared to the human sequences, human and mouse miR-204 are identical, while human and mouse miR-211 differ by only one nucleotide, distal to the seed sequence. Furthermore, miR-204 and miR-211 are essential for the development and maturation of the eye, as miR-204 and miR-211 interact with ophthalmic-developmental transcription factors such as PAX6 (22), MEIS2 (23), and MITF (24). miR-204 was reported to be responsible for an inherited retinal dystrophy with lens and iris manifestations, underscoring its importance in ophthalmic development, and is one of the first reported miR-associated genetic diseases (25). miR-204 and miR-211 are also important for the maintenance of the epithelial properties of the RPE and for cone photoreceptor function (26, 27). Notably, their expression was shown to be directly light-regulated, with increased expression in the light and decreased expression in the dark (28). Finally, the two miRs have been reported to target different steps of lysosomal processing, including regulation of expression of the ezrin protein by miR-211 (29) and of the Rab22a protein by miR-204 (30). Altogether, these previous studies indicate that miR-204 and miR-211 each play a critical role in the maintenance of RPE physiology and support of retinal health.

The molecular mechanisms through which miR-204 and miR-211 exert their influence in the RPE specifically are less well understood. Previous studies were conducted with single KOs of either miR-204 or miR-211; however, because these miRs share the same seed sequence and are nearly homologous, differing by only one nucleotide, a KO of one of these miRs may be compensated by the other. Furthermore, miR-204 and miR-211 have thus far been studied in the eye as single global KO mice, but not in tissue- or cell-specific KOs. Considering their importance in ophthalmic development, the RPE-specific role of the miRs in normal physiology has yet to be completely defined. Thus, both spatial and temporal control of conditional deletion of both miR-204 and miR-211 could provide valuable insights into their roles in the RPE and retina.

## Results

### Expression of miR-204 and miR-211 in murine and human retinas

**Figure 1:**
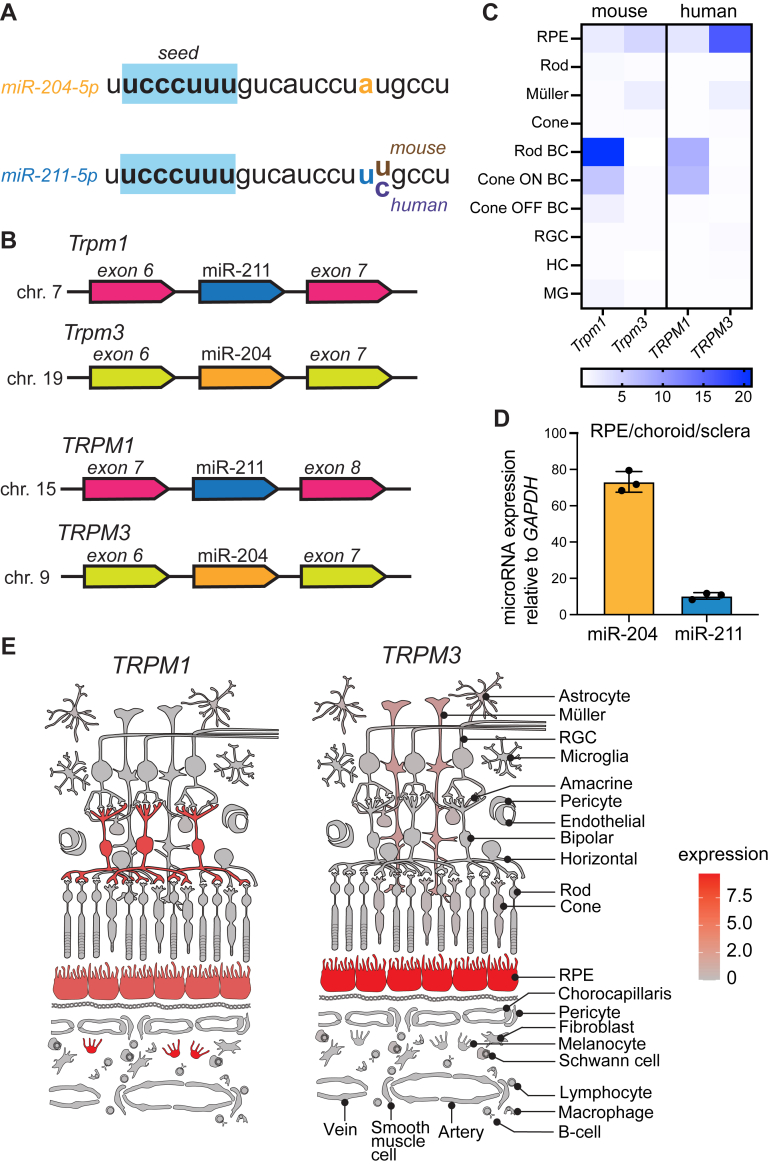
**Expression of microRNA-204 and microRNA-211 in the mouse and human eye.***A*, sequences of miR-204-5p (*upper*) and miR-211-5p (*lower*). The common seed sequence is highlighted in *blue*. A difference between miR-204 and miR-211 is highlighted as *yellow versus blue*, respectively. A difference between mouse and human miR-211 is highlighted as *brown versus purple*, respectively. *B*, organization of miR-204 and miR-211 loci within intronic regions of the genomes of mice (*upper*) and humans (*lower*). *C*, single-cell RNA expression patterns of genes containing miR-204 and miR-211 in mice and humans. *D*, qRT-PCR of miR-204 and miR-211 in RPE/choroid/sclera RNA extracted from C57BL/6J WT animals (n = 3 mice, three technical replicates), normalized to GAPDH. *E*, expression of *TRPM1* and *TRPM3* in human single-cell RNA expression sequencing, adapted from Spectacle RetinaCartoon (34). miR, microRNA; qRT-PCR, quantitative reverse transcription PCR; RPE, retinal pigment epithelium.

The two homologous miRs, miR-204 and miR-211, have high sequence identity. In the mouse, miR-204 and miR-211 differ by one nucleotide at position 17, while the seed sequences at positions 2 to 7 are conserved (Fig. 1*A*). Between the mouse and human sequences, the miR-204 sequence is completely conserved, while the mouse and human sequences differ by one nucleotide at position 18 (Fig. 1*A*). Their homology extends to their immediate genomic context. miR-204 and miR-211 are found within an intronic sequence of *Trpm3* and *Trpm1* in the mouse genome, respectively, and are found in homologous regions of *TRPM3* and *TRPM1* in the human genome; indeed, the coding sequence of exon 8 of human *TRPM1* is homologous to exon 7 of mouse *Trpm1*, and the coding sequence of exon 6 of human *TRPM3* is homologous to exon 6 of mouse *Trpm3* (Fig. 1*B*). Because the miRs are located in the intronic sequences of their respective transient receptor potential (TRP) channels, miR and TRP channel expression have been reported to be regulated cotranscriptionally (22, 31, 32). Due to polyA-RNA selection in most single-cell RNA-sequencing library preparations, many miRs are not captured. However, focusing on expression of TRPM1 and TRPM3 should capture the expression pattern of their resident miRs. Thus, we examined the expression of *TRPM1* and *TRPM3* in the retina *via* reanalysis of existing single-cell RNA sequencing datasets (33, 34). *TRPM1* is predominantly expressed in mouse and human RPE and bipolar cells, while *TRPM3* is expressed in mouse and human RPE and Müller glia (Fig. 1, *C* and *E*). Finally, miR-204 and miR-211 were confirmed to be expressed in a similar pattern in murine RPE by quantitative PCR (qPCR) (Fig. 1*D*).

### Generation of miR-204 and miR-211 conditional KO animals

**Figure 2:**
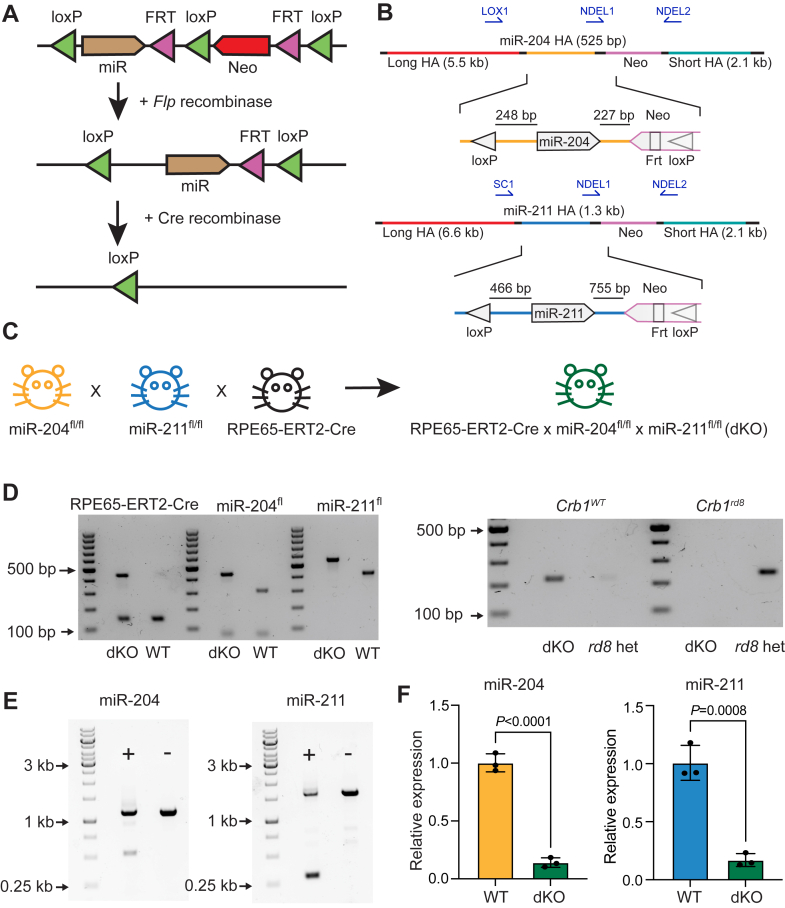
**Generation and characterization of miR-204 and miR-211 conditional-KO mice.***A*, general knock-in strategy. A cassette with loxP sites flanking the miR sequence of interest along with a selectable neomycin (Neo) marker is knocked in to the endogenous locus. After successful integration, the Neo cassette is removed *via* introduction of a *Flp* recombinase. Upon subsequent introduction of a *Cre* recombinase, the floxed miR cassette is removed. *B*, targeting strategy for miR-204 and miR-211. Location for genotyping and recombination assessment PCRs are indicated (see Table S1). HA, homology arm. *C*, breeding strategy for double knockout (dKO) mice. The two miR conditional-KO animals were crossed to the RPE65-ERT2-Cre mouse to generate a triple cross for conditional RPE-specific knockout of miR-204 and miR-211 upon administration of tamoxifen. *D*, *left*: PCR genotyping of genomic DNA for the RPE65-ERT2-Cre, miR-204, and miR-211 alleles; right: PCR genotyping of genomic DNA for the *Crb1 rd8* mutation. *E*, PCR analysis of miR deletion in dKO animals 1-month post tamoxifen induction. Lower band indicates successful recombination. *F*, qRT-PCR of miR-204 (*left*) and miR-211 (*right*) in dKO animals 1-month post-tamoxifen induction (n = 3 mice, three technical replicates), normalized to dKO animals (n = 3 mice, three technical replicates) treated with corn oil vehicle. Statistical significance calculated by unpaired *t* test. dKO, double knockout; miR, microRNA; qRT-PCR, quantitative reverse transcription PCR; RPE, retinal pigment epithelium.

To assess the role of miR-204 and miR-211 in normal retinal physiology without confounders from eye development or miR-204 and miR-211 effects in other tissues, we created conditional KO animals for miR-204 and miR-211 to spatially and temporally control depletion of the miRs. *Via* standard genetic engineering techniques, we created knock-in mice with neomycin-selectable cassettes, with each miR flanked by loxP sites, into their corresponding endogenous loci (Fig. 2, *A* and *B*). Upon induction of Cre recombinase expression, Cre-mediated excision removes DNA between the loxP sites for a Cre-dependent KO (Fig. 2*A*). We crossed the newly generated floxed miR-204 and miR-211 mice, miR-204^fl/fl^ and miR-211^fl/fl^, with the tamoxifen-inducible RPE65-ERT2-Cre mice to generate double knockout (dKO) mice (Fig. 2*C*) (35). This approach allows for RPE-specific and temporally controlled conditional KO of these two miRs, as this RPE Cre driver line is highly efficient and specific. We confirmed that these alleles were detectable by PCR genotyping, and that the founders did not carry the *Crb1 rd8* mutation commonly found in C57BL/6 lines (Fig. 2*D*) (36). Our mice were homozygous for the less active RPE65 M450 allele (Fig. S1) (37). To assay successful recombination, we performed simultaneous DNA and RNA extraction from posterior eyecups of dKO *versus* control animals. PCR of genomic DNA showed that tamoxifen induction of Cre in dKO mice led to successful deletion of the floxed miR alleles (Fig. 2*E*). Of note, the lysis buffer used for nucleic acid extraction leads to contamination of the RPE isolation with sclera, choroid, and residual neural retina, explaining the presence of PCR products from unrecombined alleles. At the RNA level, tamoxifen induction led to significant knockdown of the miR-204 (7.12-fold, *p* < 0.0001) and miR-211 (5.89-fold, *p* = 0.0008) (Fig. 2*F*).

### RPE-specific KO of miR-204 and miR-211 leads to slow retinal degeneration

**Figure 3:**
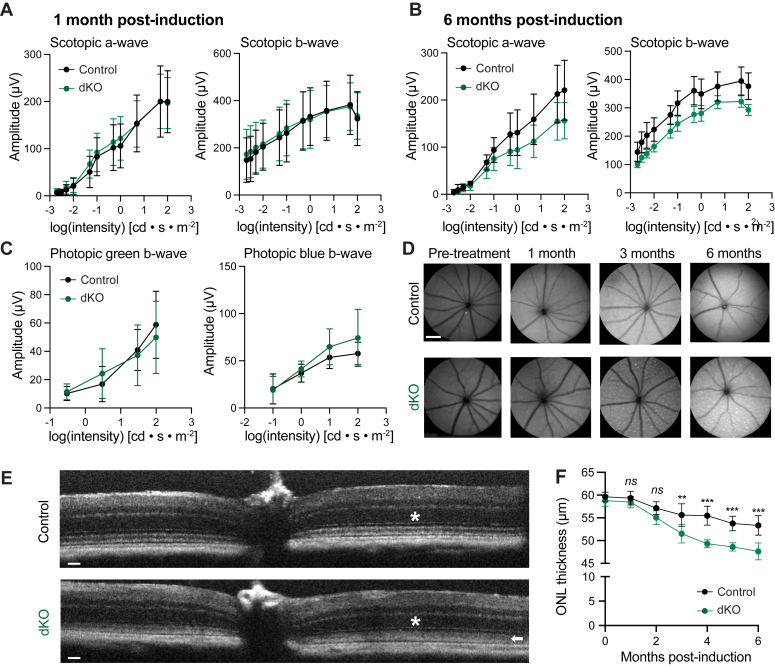
**miR-204 and miR-211 dKO mice exhibit retinal degeneration.***A*, assessment of scotopic full-field flash electroretinography (ERG) a-wave (*left*) and b-wave (*right*) responses in dKO mice (*green*, n = 16) *versus* control mice (*black*, n= 16), 1 month after tamoxifen induction. *B*, assessment of scotopic full-field flash ERG a-wave (*left*) and b-wave (*right*) responses in dKO mice (*green*, n = 12) *versus* control mice (*black*, n= 6), 6 months after tamoxifen induction. *C*, assessment of photopic full-field flash ERG green response (*left*) and blue response (*right*) in dKO mice (*green*, n = 10) *versus* control mice (*black*, n= six), 6 months after tamoxifen induction. *D*, scanning laser ophthalmoscopy (SLO) autofluorescent fundus imaging of control mice (*upper*) and dKO mice (*lower*), at various periods after tamoxifen induction. Scale bar represents 500 μm. *E*, representative spectral-domain optical coherence tomography (OCT) images of control and dKO mice at 6 months postinduction. *Asterisk* indicates outer nuclear layer (ONL), *arrowhead* indicates loss of definition in dKO mice between IS/OS junction and RPE. Scale bar represents 50 μm. *F*, quantification of ONL thickness by OCT in control mice (*black*, n ≥ 7 mice) and dKO mice (*green*, n ≥ 9 mice). For all plots, statistical significance calculated by multiple unpaired *t* test with two-stage step-up FDR (Benjamini, Krieger, and Yekutieli). dKO, double knockout; FDR, false discovery rate; IS/OS, inner segment/outer segment; miR, microRNA.

We first characterized our dKO animals *via* noninvasive longitudinal imaging and electrophysiological techniques. To avoid potential effects of miR deletion on development, we induced Cre expression at postnatal day 28. We utilized Cre-negative animals treated with tamoxifen as controls, as there was no difference in retinal phenotypes between Cre-negative animals treated with tamoxifen *versus* Cre-positive animals treated with vehicle (Fig. S2, *A* and *C*). We also noted no Cre-line independent phenotypes in Cre-only animals without conditional miR alleles after tamoxifen induction (Fig. S2*B*). One-month after induction, there was no difference in the scotopic full-field flash electroretinogram (ERG) a- or b-wave amplitudes (Fig. 3*A*). At 6-months postinduction, there was a trend toward suppression of the scotopic ERG a-wave and concomitant decrease in the scotopic ERG b-wave, though this was not significant by two-way ANOVA (*p* = 0.2352 and *p* = 0.1239 for a- and b-wave, respectively, Fig. 3*B*). At 6 months after induction, we noticed no difference in the photopic full-field flash ERG for green or blue cones (Fig. 3*C*). These data indicate that 6 months of miR ablation did not lead to a functional deficit in rod or cone photoresponses. We then assessed structural and anatomical changes by performing autofluorescence-scanning laser ophthalmoscopy (SLO) and noted subretinal hyperautofluorescent foci as early as 2 months post induction in dKO mice, but not in control animals, potentially indicating that ongoing degenerative and inflammatory processes were occurring in the RPE and subretinal space (Fig. 3*D* and S3). Lastly, we imaged the mouse eye with spectral-domain optical coherence tomography (OCT) to evaluate the anatomical structures of the retinal layers, which showed a 10.7% loss of the ONL by 6 months post induction (*p* = 0.000032) (Fig. 3*E*). Thus, the dKO animals exhibited a slow but progressive loss of the ONL, as well as a loss of definition of the inner segment/outer segment (IS/OS) junction and RPE layers, indicative of ongoing photoreceptor death and loss (Fig. 3, *E* and *F*).

**Figure 4:**
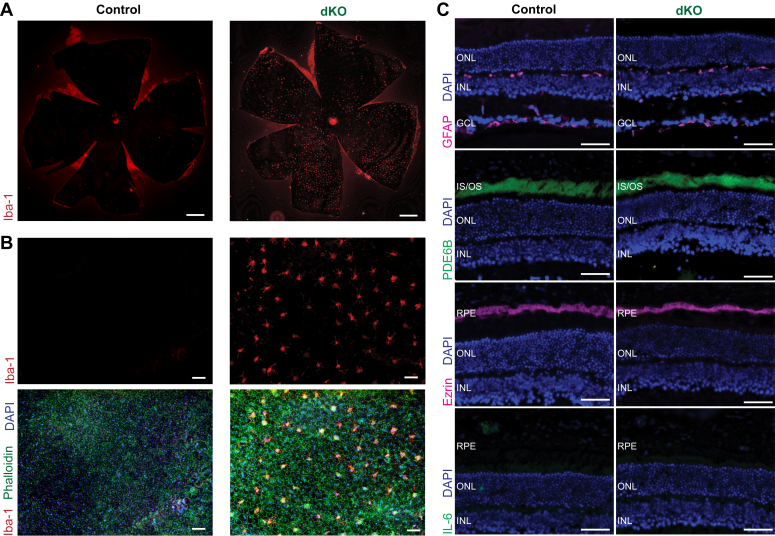
**Retinal inflammation in dKO mice.***A*, whole RPE flatmounts of control mice (*left*) and dKO mice (*right*), 6 months post-induction, stained with Iba-1 (*red*). Scale bar represents 500 μm. *B*, magnified RPE flatmounts of control mice (*left*) and dKO mice (*right*), stained with Iba-1 (*red*), phalloidin (*green*), and DAPI (*blue*). Scale bar represents 50 μm. *C*, cryosection immunostaining of 6-months postinduction control mice (*left*) and dKO mice (*right*), stained with GFAP, PDE6B, Ezrin, IL-6, and DAPI. Scale bar represents 50 μm. DAPI, 4′,6-diamidino-2-phenylindole; dKO, double knockout; IL-6, interleukin 6; RPE, retinal pigment epithelium.

Because we had observed hyperautofluorescent foci upon SLO imaging of the dKO animals, we hypothesized that this signal resulted from inflammatory processes as had been previously shown in other inflammatory retinal disease states (38, 39). Therefore, we created RPE flatmounts from 6-months postinduction dKO and control mice and evaluated them for the presence of infiltrating immune cells. We noted Iba-1-positive cells in the subretinal space in dKO mice, but not in control mice, indicating the pathogenic finding of immune cell recruitment to the subretinal space (Fig. 4, *A* and *B*) (40). We also assessed the expression of RPE, retinal, and inflammatory markers such as GFAP, PDE6B, ezrin, and interleukin 6 in cryosections of 6-months postinduction control and dKO mice and found no significant differences (Fig. 4*C*).

### Retinas and RPE from dKO mice exhibit degeneration and structural perturbations

**Figure 5:**
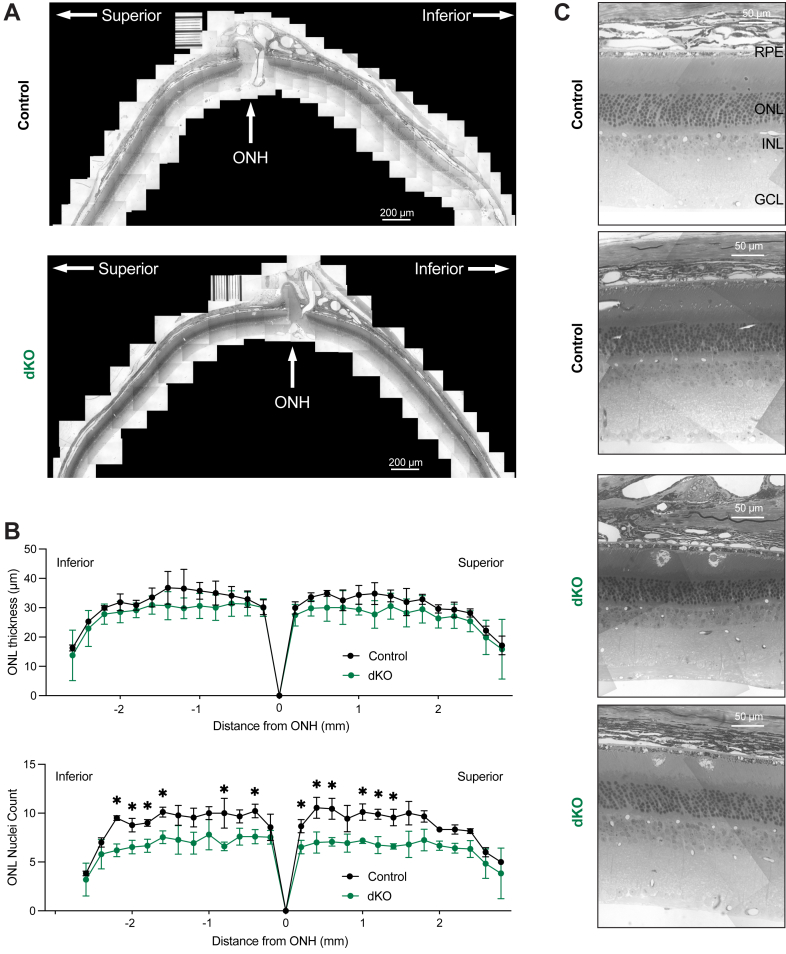
**Light microscopy of dKO mice reveals photoreceptor degeneration and subretinal pathology.***A*, representative stitched-light microscopy images of retinas from control mice (*upper*) and dKO mice (*lower*), 6 months post-induction. ONH, optic nerve head. Scale bars represent 200 μm. *B*, spider plot of ONL thickness (*upper*) and ONL nuclei count per column (*lower*) at various distances (μm) from the ONH, quantified from stitched-light microscopy images from control mice (*black*, n = 3 mice) and dKO mice (*green*, n = 5 mice). Statistical significance calculated by multiple unpaired *t* test with two-stage step-up FDR (Benjamini, Krieger, and Yekutieli). ONL thickness was not significant, ONL nuclei count was significant at multiple distances. *C*, magnified light-microscopy images reveal subretinal pathology in dKO animals. GCL, ganglion cell layer; INL, inner nuclear layer; ONL, outer nuclear layer; RPE, retinal pigment epithelium. Scale bars represent 50 μm. dKO, double knockout; FDR, false discovery rate.

We performed stitched light microscopy of fixed whole retinas to assess global retinal structure (Fig. 5*A*). The retinas from 6-months postinduction dKO mice exhibited decreased ONL thickness and decreased nuclei counts per column (Fig. 5, *B* and *C*). While there was less difference in the total ONL thickness measured by microscopy *versus* the *in situ* measurements performed by OCT, the significantly lower number of nuclei in the ONL in dKO mice at multiple points among the superior-inferior axis indicates a significant loss of photoreceptors. We also noted subretinal infiltrates and cells in dKO mice, but not in control mice, suggestive of either subretinal deposits or immune cells that had migrated into the subretinal space (Fig. 5*C*).

**Figure 6:**
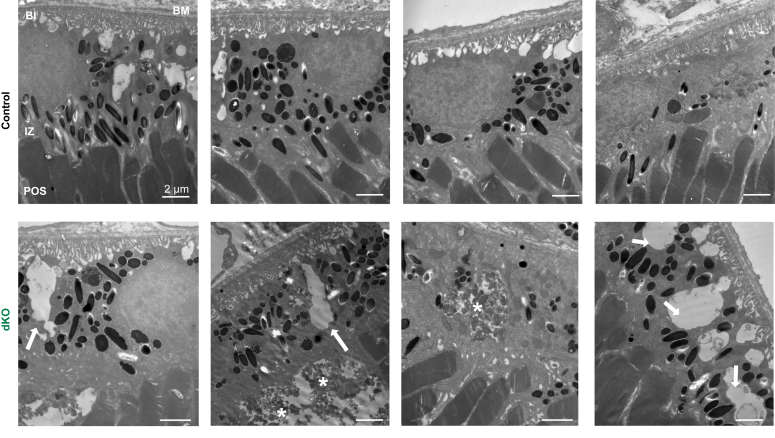
**Transmission electron microscopy (TEM) of dKO mice reveals RPE and outer segment pathology.** The RPE and retinas from dKO mice displayed basolateral vacuolization (*arrowheads*) and subretinal pathology (*stars*). Scale bars represent 2 μm. Bl, basal infoldings; BM, Bruch’s membrane; dKO, double knockout; IZ, interdigitation zone; POS, photoreceptor outer segments; RPE, retinal pigment epithelium.

To analyze the ultrastructural morphology of the RPE and photoreceptors in the same dKO animals 6 months after induction, we examined the RPE under transmission electron microscopy (EM). In control animals, we observed well-laminated photoreceptor outer segments and morphologically regular RPE organelles (Fig. 6). In contrast, dKO mice exhibited subretinal pathology (stars), attributable to either infiltrating macrophages/microglia or subretinal deposits, as well as basolateral vacuolization (arrows) (Fig. 6). The subretinal pathology was not observed in control animals, while some vacuoles were observed in control animals; the vacuoles were larger, more pronounced, and in some cases, nearly spanned the whole thickness of the cell from the basal side to the apical side.

### Single-cell RNA sequencing of dKO retinas reveals subtle transcriptomic changes

**Figure 7:**
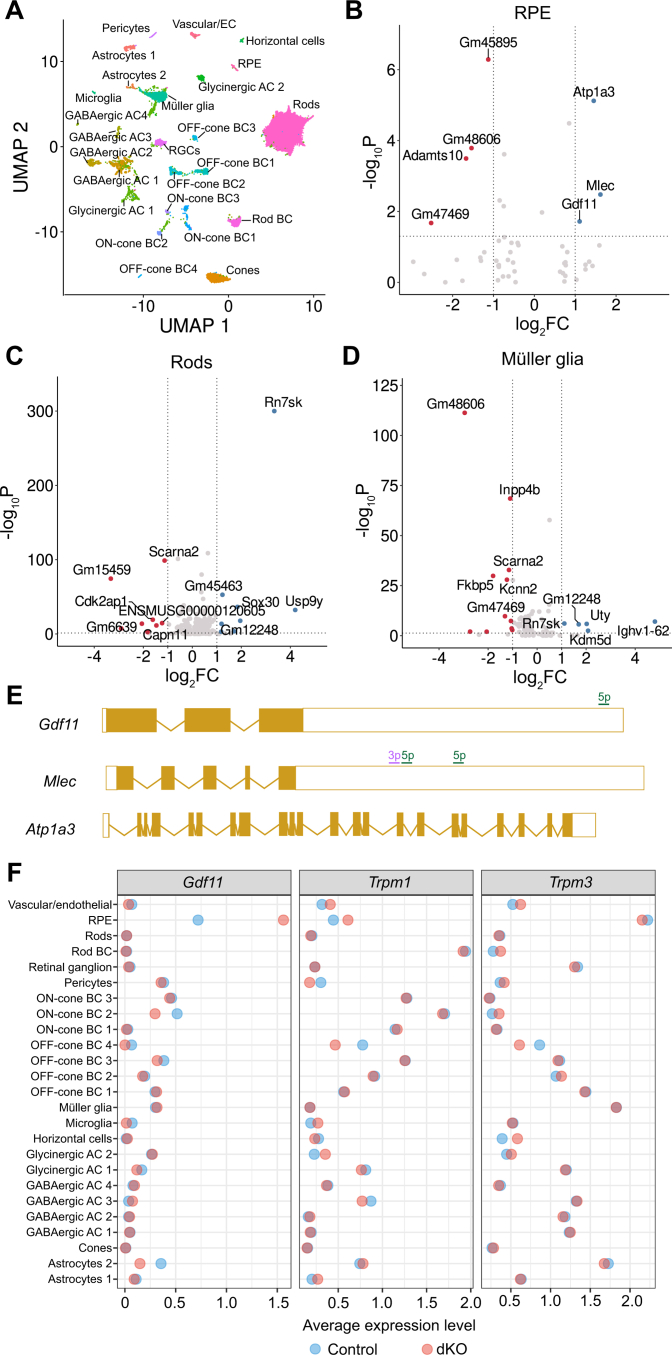
**Single-cell RNA sequencing of retina and RPE in dKO and control animals.***A*, merged uniform-manifold approximation and projection (UMAP) clustering from analysis of retinas from four control and five dKO mice, totaling 31,089 cells. *B*, volcano plots of differentially expressed genes in RPE cells (417 cells). *C*, volcano plots of differentially expressed genes in rod photoreceptors (15,414 cells). *D*, volcano plots of differentially expressed genes in Müller glia (2126 cells). *E*, MiR-204/211 family binding sites in upregulated mRNA from the RPE. Exons are shaded, while 5′- and 3′-UTRs are unshaded. 5p binding sites are indicated in *green*, 3p binding sites are indicated in *purple*. *F*, lollipop plots demonstrate significant upregulation of *Gdf11* (*left*) in dKO RPE (*red*) *versus* control RPE (*blue*). Lollipop plots demonstrate unaltered *Trpm1* (*middle*) and *Trpm3* (*right*) expression in dKO (*red*) *versus* control (*blue*) retinas. dKO, double knockout; GDF11, growth differentiation factor 11; RPE, retinal pigment epithelium.

To identify dysregulated pathways and potential miR targets in the RPE and retina, we performed single-cell RNA-sequencing of RPE and retinas, analyzing 31,089 cells from five dKO mice and four control mice (Fig. 7*A*) 6 months post induction. Surprisingly, despite deletion of RPE miR-204 and miR-211, minimal transcriptomic changes were found in the RPE (417 cells, Fig. 7*B*). In RPE cells from dKO mice, there were three significantly upregulated genes, *Gdf11, Mlec*, and *Atp1a3*, along with four significantly downregulated genes, *Gm47469, Gm48606, Gm45895*, and *Adamts10* (Fig. 7*B*). Two of the upregulated genes, *Gdf11* and *Mlec*, contained sequences in the 3′-UTR that could bind the miR-204/miR-211 5p or 3p seed sequences, while *Atp1a3* did not contain any canonical miR-204/miR-211 5p or 3p binding sites (Fig. 7*E*). Thus, *Gdf11* and *Mlec* may be *bona fide* primary targets of miR-204 and miR-211 mRNA regulation, while *Atp1a3* upregulation may be the result of a secondary process. Next, we analyzed the rod photoreceptors and Müller glia, as RPE-specific miR-204 and miR-211 deletion led to rod photoreceptor degeneration. Again, despite ongoing degenerative and inflammatory processes, a relatively small number of genes were differentially expressed in rod photoreceptors (15,414 cells, Fig. 7*C*) and Müller glia (2126 cells, Fig. 7*D*). Intriguingly, two long noncoding RNAs (lncRNAs), *Gm48606* and *Gm47469*, were both downregulated in RPE and Müller glia (Fig. 7, *B* and *D*), while *Rn7sk* was upregulated in both rod photoreceptors and Müller glia (Fig. 7, *C* and *D*). Lastly, we confirmed that deletion of miR-204 and miR-211 did not significantly affect the expression of *Trpm1* and *Trpm3* in our sequencing data, as compared to *Gdf11* (Fig. 7*F*).

## Discussion

In this study, we investigated whether the conditional deletion of two miRs highly expressed in the RPE resulted in altered RPE physiology and subsequent retinal degeneration. The two miRs, miR-204 and miR-211, were previously shown to maintain numerous eye functions in the RPE, retina, and other ocular tissues such as the ciliary body and iris. However, because other studies performed single, global KOs of only one or the other of these two highly homologous miRs, those studies may have been confounded in the interpretation of their roles in maintaining normal physiological function by the effect on the developing eye and RPE, or by compensation from the homologous miR. We hypothesized that deletion of these two miRs together specifically in the RPE after development would alter RPE physiology and lead to subsequent neural retinal degeneration.

We demonstrated conditional deletion of miR-204 and miR-211 in the RPE in the developed retina after administration of tamoxifen to the RPE65-driven Cre dKO animals at postnatal day 28. After miR-204 and miR-211 deletion in the RPE, we observed a progressive retinal degeneration. Even though the two miRs are expressed in multiple cell types throughout the eye, RPE-specific deletion primarily led to photoreceptor death. This is further evidence of the critical role the RPE plays in supporting photoreceptor health and function, as dysfunction of the RPE propagated to the photoreceptors. Visual function, measured by ERG, was slightly affected by the degeneration, but was accompanied by more severe anatomical degeneration, quantified by OCT and tissue histology. It is likely that if the degeneration proceeded for a longer period of time, we would observe a larger decline in the electrophysiology in rods and perhaps cones. These changes were evident at an ultrastructural level, as the organization and structure of the RPE and RPE-photoreceptor interface was altered in EM images collected from dKO mice. We also observed a chronic inflammatory process in the RPE-photoreceptor interface, presumably secondary to an inflammatory or degenerative signal from stressed photoreceptors which recruited Iba-1-positive macrophages/microglia into the subretinal space. However, another possibility is that miR-204 and miR-211 in the RPE could directly promote an antiinflammatory environment.

We performed single-cell RNA sequencing of the RPE and retina to determine mechanisms of RPE dysfunction, photoreceptor damage, and potential miR-regulated targets in the RPE. However, in our single-cell RNA sequencing, we were surprised that in a degenerative state with inflammatory immune cell recruitment and photoreceptor death, we recovered unexpectedly few differentially expressed genes. It is conceivable that this result could have been due to a relatively lower number of RPE cells in the tissue sample, leading to lower power. However, even the rod photoreceptors, which are the most abundant cell type in the retina and in the data set, and the ones most heavily affected by the degeneration, showed relatively few differentially expressed genes. The two potential miR-regulated protein-coding genes in the RPE were *Gdf11* and *Mlec*. *Gdf11* produces the protein growth differentiation factor 11 (GDF11), a member of the transforming growth factor beta superfamily of secreted signaling proteins. GDF11, also known as bone morphogenic factor 11, was previously shown to regulate retinal progenitor differentiation in the developing retina (41). As a secreted factor, GDF11 could be an intercellular communication mechanism in this degenerative process, or a chemotactic signal for infiltrating immune cells. However, proteins from dying photoreceptors are sufficient to recruit immune cells into the subretinal space, implying that GDF11 may play another role in this pathology, as reports from other organ systems indicate that GDF11 is pleiotropic and can promote muscle and neural regeneration. The other differentially regulated gene, *Mlec*, encodes malectin, an endoplasmic reticulum-expressed carbohydrate protein shown to bind N-glycosylated proteins (42). Altered glycosylation of extracellular proteins may also play a role in disrupting RPE-photoreceptor crosstalk or in regulating endoplasmic reticulum stress in altered proteostasis (43). Many of the additional genes identified as being differentially regulated in all clusters were lncRNAs, but relatively little is known about the role of lncRNAs in the cell biology of the RPE and retina (44). We also examined the Müller glia compartment, as this cell type also closely supports the neural function of the retina. It is known that Müller glia and photoreceptors can directly communicate, such as through the alternative visual cycle (45), though the exact mechanisms through which this occurs are poorly understood. This interaction among the three cell types could explain why some genes were apparently coregulated in the RPE, photoreceptors, and Müller cells. These parallel changes in lncRNAs are more likely to be a common response to degeneration, rather than deregulation post KO, as the differential expression in multiple cell types points to an association with a pathological state.

Why, then, are these miRs so highly expressed in the RPE and retina, when the effect of their deletion on gene regulation was relatively modest? One potential explanation is that lncRNAs, other noncoding RNAs, and miRs interact cooperatively to regulate cellular function in a way that could be resistant to perturbation. It is also possible that miR expression in higher animals in normal physiology merely fine-tunes gene expression, and loss of this adjustment mechanism results in a very gradual degradation of the tissue. In contrast with less complex organisms such as *C. elegans*, higher order mammals such as mice have evolved additional regulatory networks and modules that reduce the need for miR regulation of gene expression. While miRs have been shown to play a key role during development and in states such as infection and cancer (46), they are potentially less critical within a homeostatic context. Another possibility is that there are additional unidentified members of the miR-204/miR-211 family, or that other miR families can regulate the target genes of miR-204 and miR-211. Alternatively, miR-204 and miR-211 loss could be alternatively compensated for by unidentified genetic mechanisms, as their deletion is seemingly tolerated relatively well in the mouse. It is also possible that while our Cre animal was highly (>99%) efficient, it may not have been 100% efficient, leading to a residual amount of miR-204 and miR-211 that maintained RPE physiology. Lastly, another potential explanation is the relative importance of the TRP ion channels TRPM1 and TRPM3, which are coregulated with miR-204 and miR-211. TRPM1 and TRPM3 have well-known roles in retinal physiology and phototransduction, as they are highly expressed in bipolar cells (BCs) and *TRPM1* mutations lead to congenital stationary night blindness, but these channels are less well characterized in the RPE (47). In one study, TRPM3 deletion in the murine retina did not lead to a change in scotopic or photopic ERG, though animals in this study were only studied between postnatal days 21 to 50 (48). While TRPM1 and TRPM3 have not been definitively linked to specific roles in the RPE, it could be that it is the TRP channels that are required by the RPE, while the miRs play a lesser role. In our study, *Trpm1* and *Trpm3* expression were unchanged in our RNA-seq dataset, so the retinal degenerative phenotype can be linked conclusively to the loss of miR-204 and miR-211; however, this does not preclude a larger role for the TRP channels in the RPE.

To summarize, the conditional deletion of miR-204 and miR-211 in the adult murine RPE resulted in progressive retinal degeneration of photoreceptors. Thus, deletion of miRs in the adult eye specifically in RPE can lead to the loss of photoreceptors, which has only previously been shown to occur in global KO conditions. This indicates that miR-204 and miR-211 play a role in maintaining normal physiology apart from their role in regulating ophthalmic development. While the major targets and mechanisms by which RPE dysfunction resulted in photoreceptor cell dysfunction and death remain incompletely resolved, this work highlights how the intertwined RPE-photoreceptor system works cooperatively as a functional unit and how perturbation of the RPE leads to photoreceptor death. Further work may elucidate the exact mechanisms by which the RPE, photoreceptors, and Müller glia communicate their homeostatic states. Additionally, further technological progress in enabling higher resolution single-cell analysis of both coding and noncoding RNA in the RPE could more completely define the roles of these two miRs in the RPE and for maintaining cellular health.

## Experimental procedures

### Animals

C57BL/6J (“WT”) mice were purchased from the Jackson Laboratory (#000664). RPE65-ERT2-Cre mice were generated at the University of California, Irvine (35), and are also available from the Jackson Laboratory (#035973). The miR-204^fl/fl^ (Jackson Laboratory, #039332) and miR-211^fl/fl^ (Jackson Laboratory, #039333) mouse lines were constructed under contract by InGenious Targeting Laboratory with standard techniques and deposited at the Jackson Laboratory. C57BL/6 bacterial artificial chromosome (BAC) clones were subcloned *via* homologous recombination into targeting vectors with loxP sites flanking the miRs and a flippase recognition target (FRT)-flanked reverse strand neomycin cassette downstream of the miRs (sequences in Tables S2 and S3). The targeting vector was then linearized before electroporation into Flp-embryonic stem cells. Electroporated embryonic stem cells were then microinjected into BALB/c blastocysts and mice with high levels of chimerism were then mated to C57BL/6 mice to produce germline neomycin-deleted offspring. The locus was then sequenced and founders were screened for the *Crb1*^*rd8*^ mutation.

All mouse strains were housed in the vivarium at the University of California, Irvine, where they were maintained on a normal mouse chow diet and a 12 h/12 h light/dark cycle. Cre induction was performed by five sequential days of injection of tamoxifen (Sigma-Aldrich #T5648) dissolved in corn oil at 20 mg ml^–1^ and administered intraperitoneally at 75 mg kg^–1^. All animal procedures were approved by the Institutional Animal Care and Use Committee of the University of California, Irvine. All animal procedures were conducted in accordance with the National Institutes of Health guidelines for the care and use of laboratory animals, and with the Association for Research in Vision and Ophthalmology Statement for the Use of Animals in Ophthalmic and Visual Research.

### PCR

For routine-genotyping PCR, tissue from mouse ear punches was lysed overnight at 55 °C in DirectPCR Ear Lysis Buffer (Viagen Biotech #401-E) supplemented with proteinase K (Viagen Biotech #501-PK). Proteinase K was deactivated by incubation at 85 °C for 45 min. End point PCR was conducted with Promega GoTaq G2 master mix (M7822, Promega). Primers and cycling conditions are indicated in Table S1.

### RPE dissociation and genomic DNA and RNA isolation

Mouse eyes were dissected under a light microscope to separate the posterior eyecup (containing the RPE, choroid, and sclera) from the neural retina and anterior segment. Retinas and RPE were placed into QIAzol reagent and nucleic acids isolated with a miRNeasy Mini kit (Qiagen #217004), AllPrep Micro DNA/RNA kit (Qiagen #80284), or a DNeasy Blood and Tissue kit (Qiagen #69504).

### qPCR

miRNA qPCR was conducted with the Qiagen miScript II kit (Qiagen #218073), according to the manufacturer instructions. Briefly, total RNA isolated with the miRNeasy Mini kit was quantified by Nanodrop UV absorbance, and input for the reverse transcription reaction was normalized across samples. Complementary DNA was then produced in miScript HiFlex buffer and diluted with water before qPCR. Reactions for qPCR were set up with 2× QuantiTect SYBR Green PCR Master Mix (Qiagen), along with the 10× Universal Primer and either miR-204 (Qiagen #MS00032557) or miR-211 primers (Qiagen #MS00001897) predesigned by Qiagen to discriminate between the two miRs. In a control reaction, GAPDH primers were utilized. Reaction mixtures were then dispensed into a 384-well plate (Bio-Rad) and processed on a CFX384 Touch Real-Time PCR system (Bio-Rad) under the following conditions: 95 °C, 15 min; then 40 cycles of 94 °C, 15 s; 55 °C, 30 s; 72 °C, 30 s; and a 65 °C to 95 °C melt curve. Data were analyzed *via* the ΔΔCt method.

### Light and electron microscopy

Mice were euthanized under anesthesia, and tissues were fixed by intracardiac perfusion with 2% formaldehyde and 2.5% glutaraldehyde in 0.1 M Na phosphate buffer, pH 7.2. The nasal and temporal hemispheres of each eyecup were fixed additionally in 1% osmium tetroxide dissolved with 0.1 M Na phosphate, then dehydrated in a graded series of alcohols. The temporal hemispheres were embedded in an Epon/Araldite mixture (5 parts/3 parts) for light microscopy. The nasal hemispheres were cut into quadrants and embedded in Araldite 502 (Ted Pella) for EM. Oculus dexter (OD, right eye) was prepared for EM, and oculus sinister (OS, left eye) was prepared for light microscopy. Ultrathin sections for EM and semithin sections for light microscopy were cut on a Leica Ultracut ultramicrotome. The sections for EM were collected on copper grids and stained with UranyLess (#22409 EMS) and lead citrate (#22410 EMS) before viewing on a JEOL JEM—100CX II Electron Microscope. Electron microscopic images were acquired from the inferior-nasal quadrant sections. Light microscopy sections were viewed on a Zeiss Axiophot Microscope and acquired with RSImage+. Images were analyzed and stitched using ImageJ (https://imagej.net/ij/index.html) and TrakEM (https://github.com/trakem2/TrakEM2).

### Electroretinography

Prior to ERG recording, mice were dark adapted overnight. Under a red safety light, mice were anesthetized by intraperitoneal administration of a cocktail consisting of 20 mg ml^–1^ ketamine and 1.6 mg ml^–1^ xylazine in PBS at a dose of 100 mg kg^–1^ of ketamine and 8 mg kg^–1^ of xylazine. Their pupils were dilated with topical administration of 1% tropicamide ophthalmic solution (Akorn; 17478-102-12) and 10% phenylephrine ophthalmic solution (MWI Animal Health #054243), followed by hypromellose (Akorn; 9050-1) for hydration. Each mouse was placed on a heated Diagnosys Celeris rodent-ERG device (Diagnosys LLC). Ocular stimulator electrodes were placed on the corneas, the reference electrode was positioned subdermally between the ears, and a ground electrode was placed in the rear leg. For single-flash scotopic ERG recording, the duration of the green-light flash stimuli (from 20 μsec to 1 msec) was adjusted to provide a range of illumination intensities from −3.7 to 2.3 log (cd·s m^–2^). For each intensity, 3 to 20 recordings were made at sufficient intervals between flash stimuli (from 3 to 90 s) to allow recovery from any photobleaching effects. The photopic ERG recordings were performed after bleaching at 1.4 log (cd·s m^–2^) for 10 min. For photopic ERG, the cone response was measured at four different light intensities (−0.7–2.3 log (cd·s m^–2^)) in the presence of rod-desensitizing white-light background. After recording, the mice were placed on a heating pad and anesthesia was reversed with intraperitoneal atipamezole (2.5 mg kg^–1^, MWI Animal Health #032800). Data were analyzed with Espion V6 software (Diagnosys LLC, https://info.diagnosysllc.com/software).

### OCT and SLO

Mice were anesthetized by intraperitoneal administration of a cocktail consisting of 20 mg ml^–1^ ketamine and 1.60 mg ml^–1^ xylazine in PBS at a dose of 100 mg kg^–1^ of ketamine and 8 mg kg^–1^ of xylazine. Their pupils were dilated with 1% tropicamide, and the mice were placed on a warming pad. SLO images were first acquired to obtain whole-fundus images *in vivo*. A Heidelberg Retinal Angiograph II (Heidelberg Engineering) in the fluorescence mode was used to acquire the images, focused on the most concentrated autofluorescent spots in each eye, which were then analyzed qualitatively. OCT was then performed on the same animal with a Bioptigen spectral-domain OCT device (Leica Microsystems Inc). After imaging, the mice were placed on a heating pad and anesthesia was reversed with intraperitoneal atipamezole (2.5 mg kg^–1^, MWI Animal Health #032800). Four frames of OCT b-scan images were acquired from a series of 1200 a-scans. Retinal ONL thickness was measured 500 μm away from the optic nerve head in four retinal quadrants (nasal, temporal, superior, and inferior), using a ruler tool in ImageJ (NIH, v1.52a, https://imagej.net/ij/index.html). ONL thickness was averaged over the four retinal quadrants for all of the analyses.

### RPE flatmounts

Mice were euthanized by CO_2_ inhalation followed by cervical dislocation, and dissection and staining were performed as previously described (39). Briefly, the superior position of the eye was marked on the cornea with a blue Sharpie; globes were enucleated and fixed in PBS-buffered 4% paraformaldehyde (PFA) for 10 min. After fixing, eyes were washed three times in PBS for 3 min and placed on a microscope slide. Muscles, fat, and optic nerve were removed from the globe, followed by a puncture in the center of the cornea with a 25-gauge needle. Cohan-Vannas spring scissors (Fine Science Tools) were used to make four symmetric radial incisions starting from the center of the cornea and ending directly before the optic nerve head. The lens and vitreous were removed, and the eyecup was oriented with the sclera side facing up, and flattened. The corneal flaps were removed, and the superior position was marked by making a small triangular cut. The RPE-eyecup was gently peeled off the retina and flattened on a new slide and further fixed in PBS-buffered 4% PFA for 30 min. Finally, all flatmounts were washed three times in PBS for 5 min and used for immunofluorescence staining.

RPE-eyecup flatmounts were incubated in a blocking buffer consisting of 3% bovine serum albumin with 0.3% Triton X-100 in PBS for 2 h at 22 °C. Next, rabbit anti-Iba1 Ab was added at 1:1000 dilution (#019-19741, Fujifilm Wako) in blocking buffer overnight at 4 °C, in a humidified chamber. Then, the primary Ab was removed with five rinses of PBS containing 0.3% Triton X-100. Flatmounts were incubated with a secondary goat anti-rabbit IgG Alexa Fluor 594 Ab (#A11037, Invitrogen) at 1:500 dilution in blocking buffer for 2 h at 22 °C, and then rinsed four times with PBS containing 0.3% Triton X-100. The RPE-eyecup flatmounts were incubated in Phalloidin Alexa Fluor 488 (#A12379, Thermo Fisher Scientific) at 1:1000 dilution in blocking buffer for 30 min at 22 °C, and then rinsed four times with PBS containing 0.3% Triton X-100, and once with PBS. Finally, specimens were mounted with Vectashield medium with 4′,6-diamidino-2-phenylindole (DAPI) (#H-2000; Vector Lab) and protected with coverslips. Images were captured with a Z-stack mode using a Keyence BZ-X800 microscope (Keyence Corp).

### Immunohistochemistry

Mouse eye cups were fixed for 1 h in PBS containing 4% (wt/vol) PFA (Sigma-Aldrich) at room temperature. After fixation, the eye cups were incubated sequentially in PBS containing 10, 20, or 30% (wt/vol) sucrose (Sigma-Aldrich) for 30 min at room temperature. Then, the eye cups were infiltrated with a 2:1 mixture of PBS containing 30% sucrose and optimal cutting temperature compound (VWR International) and frozen with dry ice. Retinal sections were cut at a thickness of 12 μm and stored at −80 °C until use. The retinal sections were rehydrated with PBS and blocked with PBS containing 5% (vol/vol) goat serum (Thermo Fisher Scientific) and 0.2% (vol/vol) Triton X-100 (Sigma-Aldrich). After blocking, the retinal sections were incubated with the appropriate primary antibodies diluted in PBS containing 5% (vol/vol) goat serum and 0.1% (vol/vol) Triton X-100 (Sigma-Aldrich) overnight at 4 °C. The primary antibodies used for immunohistochemistry were an anti-ezrin antibody (1:400, Abcam, ab4069), an anti-GFAP antibody (1:300, Cell Signaling Technology, 3670S), an anti-IL-6 antibody (1:500, Abcam, ab233706), and an anti-PDE6B antibody (1:500, Thermo Fisher Scientific, PA1-722).The next day, the retinal sections were washed 3 times in PBS for 5 min each and then incubated with an Alexa Fluor 647–conjugated goat anti-mouse IgG (1:300, Thermo Fisher Scientific, A28181) or Alexa Fluor 488-conjugated goat anti-rabbit IgG (1:300, Thermo Fisher Scientific, A10034) for 2 h at room temperature in the dark. The retinal sections were washed 3 times in PBS for 5 min each and then mounted with VECTASHIELD Mounting Medium for imaging. Fluorescence images were acquired with a Keyence BZ-X800 All-in-One fluorescence microscope.

### Single-cell RNA-sequencing

#### Cell dissociation and fixation

Eyes were enucleated for retinal tissue isolation. After removing the anterior chamber and lens, retinal cells were dissociated using the Papain Dissociation System (#LK003153, Worthington Biochemical). In each group, two retinas from 1 mouse were combined and used as one sample. The standard dissociation protocol included with the Papain Dissociation System was used to dissociate pairs of retinas from single animals using half of the suggested volumes listed in the protocol for all steps following the initial 20-min papain digestion. The resulting single-cell pellets were resuspended in 1 ml of chilled, filter-sterilized PBS for counting before proceeding to single-cell fixation. Each retinal single-cell suspension was fixed using Parse Biosciences Evercode Cell V2 Fixation kits, following the standard provided protocol (Parse Biosciences). Each fixed, single-cell suspension was frozen in a −80 °C freezer immediately after fixation, using a Nalgene “Mr Frosty” isopropanol freezing container (Thermo Fisher Scientific, #5100-0001).

#### Library preparation and sequencing

The fixed single-cell suspensions were defrosted and barcoded using the Parse Biosciences Evercode WT v2 library preparation protocol (Parse Biosciences). For each sample, approximately 4000 cells were barcoded using the Evercode WTK v2 barcoding plate. A total of 31,089 barcoded cells were sequenced on an Illumina NovaSeq 6000 at a read depth of approximately 50,000 reads/cell. In total, eight sublibraries were constructed and sequenced. Standard quality control metrics were assessed by the UCI Genomics Research and Technology Hub following sequencing.

#### Bioinformatics

To generate gene-expression matrices, fastq files were processed and mapped to the GRCm39.109 reference genome, using the Parse Biosciences split-pipe v1.0.4 with default parameters (Parse Biosciences). The downstream analysis was performed using the R 4.2.2 and Seurat v4.3.0 R package (49). Low-quality cells with less than 200 genes were detected and all genes expressed in less than three cells were removed, leaving 31,089 cells and 37,725 genes for further analysis. Data were normalized using Seurat’s NormalizeData function with LogNormalize as the method and a scale factor equal to 10,000. Principal component (PC) analysis was performed on a submatrix of the top 2000 most variable genes, using the function FindVariableGenes from the Seurat package. The number of top PCs was evaluated by the elbow method keeping 20 PCs for clustering and data visualization. The batch effect between samples was removed using the package Harmony v0.1.1 (https://github.com/immunogenomics/harmony) (50). The cells were clustered using a shared nearest-neighbor modularity optimization-based clustering algorithm (FindClusters in the Seurat package). To visualize cells in two dimensions the Uniform Manifold Approximation and Projection was applied. The cluster-specific genes were computed using FindAllMarkers from the Seurat package, using the MAST test with the number of Unique Molecular Identifiers detected as a latent variable (51). The same test was used to compute differentially expressed genes between genotypes or treatment groups within cell clusters. Cell clusters were annotated by assessing known cell-type-specific markers. All plots were created using ggplot2 v3.4.1. Labels in the volcano plots were created using ggrepel v0.9.3. Raw and processed datasets have been deposited in the NCBI Gene Expression Omnibus data repository under accession number GSE255851.

### Analysis of preexisting single-cell RNA sequencing datasets

Comparative human and mouse single-cell RNA sequencing datasets were retrieved from a previously published study (33). BCs were classified as rod BCs, cone ON BCs, or cone OFF BCs *via* expression of *PRKC1*, *GRM6*, and *GRIK1*, respectively. An alternate human single-cell RNA-sequencing dataset encompassing the entire posterior eye was reanalyzed from a previously published study (34) utilizing the RetinaCartoon tool (https://github.com/drewvoigt10/RetinaCartoon). The RetinaCartoon tool compresses all BC cell types into one class.

### Statistical analyses

The data are presented as mean ± SD. All experiments were conducted at least twice independently. Unless otherwise stated, statistical analyses were performed using GraphPad Prism 10 (https://www.graphpad.com). ∗, *p* < 0.05; ∗∗, *p* < 0.01; ∗∗∗, *p* < 0.001; ∗∗∗∗, *p* < 0.0001; not significant (*ns*), *p* ≥ 0.05.

## Data availability

Raw and processed single-cell RNA-sequencing datasets have been deposited in the NCBI GEO data repository under accession number GSE255851.

## Supporting information

This article contains supporting information.

## Conflict of interest

KP is a consultant for Polgenix Inc and serves on the Scientific Advisory Board at Hyperion Eye Ltd. All other authors have declared that they have no conflict of interest with the contents of this article.

## References

[1] Fuhrmann S., Zou C., Levine E.M. (2014). Retinal pigment epithelium development, plasticity, and tissue homeostasis. Exp. Eye Res..

[2] Lakkaraju A., Umapathy A., Tan L.X., Daniele L., Philp N.J., Boesze-Battaglia K. (2020). The cell biology of the retinal pigment epithelium. Prog. Retin. Eye Res..

[3] Bartel D.P. (2018). Metazoan MicroRNAs. Cell.

[4] Peterson K.J., Dietrich M.R., McPeek M.A. (2009). MicroRNAs and metazoan macroevolution: insights into canalization, complexity, and the Cambrian explosion. Bioessays.

[5] Bartel D.P., Chen C.Z. (2004). Micromanagers of gene expression: the potentially widespread influence of metazoan microRNAs. Nat. Rev. Genet..

[6] Krol J., Loedige I., Filipowicz W. (2010). The widespread regulation of microRNA biogenesis, function and decay. Nat. Rev. Genet..

[7] Ha M., Kim V.N. (2014). Regulation of microRNA biogenesis. Nat. Rev. Mol. Cell Biol..

[8] Treiber T., Treiber N., Meister G. (2019). Regulation of microRNA biogenesis and its crosstalk with other cellular pathways. Nat. Rev. Mol. Cell Biol..

[9] Bartel D.P. (2009). MicroRNAs: target recognition and regulatory functions. Cell.

[10] McGeary S.E., Lin K.S., Shi C.Y., Pham T.M., Bisaria N., Kelley G.M. (2019). The biochemical basis of microRNA targeting efficacy. Science.

[11] Lee R.C., Feinbaum R.L., Ambros V. (1993). The C. elegans heterochronic gene lin-4 encodes small RNAs with antisense complementarity to lin-14. Cell.

[12] Wightman B., Ha I., Ruvkun G. (1993). Posttranscriptional regulation of the heterochronic gene lin-14 by lin-4 mediates temporal pattern formation in C. elegans. Cell.

[13] Orom U.A., Nielsen F.C., Lund A.H. (2008). MicroRNA-10a binds the 5'UTR of ribosomal protein mRNAs and enhances their translation. Mol. Cell.

[14] Valinezhad Orang A., Safaralizadeh R., Kazemzadeh-Bavili M. (2014). Mechanisms of miRNA-mediated gene regulation from common downregulation to mRNA-specific upregulation. Int. J. Genomics.

[15] Du S.W., Palczewski K. (2022). MicroRNA regulation of critical retinal pigment epithelial functions. Trends Neurosci..

[16] Sundermeier T.R., Sakami S., Sahu B., Howell S.J., Gao S., Dong Z. (2017). MicroRNA-processing enzymes are essential for survival and function of mature retinal pigmented epithelial cells in mice. J. Biol. Chem..

[17] Sundermeier T.R., Zhang N., Vinberg F., Mustafi D., Kohno H., Golczak M. (2014). DICER1 is essential for survival of postmitotic rod photoreceptor cells in mice. FASEB J..

[18] Kaneko H., Dridi S., Tarallo V., Gelfand B.D., Fowler B.J., Cho W.G. (2011). DICER1 deficit induces Alu RNA toxicity in age-related macular degeneration. Nature.

[19] Lumayag S., Haldin C.E., Corbett N.J., Wahlin K.J., Cowan C., Turturro S. (2013). Inactivation of the microRNA-183/96/182 cluster results in syndromic retinal degeneration. Proc. Natl. Acad. Sci. U. S. A..

[20] Karali M., Peluso I., Gennarino V.A., Bilio M., Verde R., Lago G. (2010). miRNeye: a microRNA expression atlas of the mouse eye. BMC Genomics.

[21] Karali M., Persico M., Mutarelli M., Carissimo A., Pizzo M., Singh Marwah V. (2016). High-resolution analysis of the human retina miRNome reveals isomiR variations and novel microRNAs. Nucleic Acids Res..

[22] Shaham O., Gueta K., Mor E., Oren-Giladi P., Grinberg D., Xie Q. (2013). Pax6 regulates gene expression in the vertebrate lens through miR-204. PLoS Genet..

[23] Conte I., Merella S., Garcia-Manteiga J.M., Migliore C., Lazarevic D., Carrella S. (2014). The combination of transcriptomics and informatics identifies pathways targeted by miR-204 during neurogenesis and axon guidance. Nucleic Acids Res..

[24] Adijanto J., Castorino J.J., Wang Z.X., Maminishkis A., Grunwald G.B., Philp N.J. (2012). Microphthalmia-associated transcription factor (MITF) promotes differentiation of human retinal pigment epithelium (RPE) by regulating microRNAs-204/211 expression. J. Biol. Chem..

[25] Conte I., Hadfield K.D., Barbato S., Carrella S., Pizzo M., Bhat R.S. (2015). MiR-204 is responsible for inherited retinal dystrophy associated with ocular coloboma. Proc. Natl. Acad. Sci. U. S. A..

[26] Wang F.E., Zhang C., Maminishkis A., Dong L., Zhi C., Li R. (2010). MicroRNA-204/211 alters epithelial physiology. FASEB J..

[27] Barbato S., Marrocco E., Intartaglia D., Pizzo M., Asteriti S., Naso F. (2017). MiR-211 is essential for adult cone photoreceptor maintenance and visual function. Sci. Rep..

[28] Krol J., Busskamp V., Markiewicz I., Stadler M.B., Ribi S., Richter J. (2010). Characterizing light-regulated retinal microRNAs reveals rapid turnover as a common property of neuronal microRNAs. Cell.

[29] Naso F., Intartaglia D., Falanga D., Soldati C., Polishchuk E., Giamundo G. (2020). Light-responsive microRNA miR-211 targets Ezrin to modulate lysosomal biogenesis and retinal cell clearance. EMBO J..

[30] Zhang C., Miyagishima K.J., Dong L., Rising A., Nimmagadda M., Liang G. (2019). Regulation of phagolysosomal activity by miR-204 critically influences structure and function of retinal pigment epithelium/retina. Hum. Mol. Genet..

[31] Deo M., Yu J.Y., Chung K.H., Tippens M., Turner D.L. (2006). Detection of mammalian microRNA expression by in situ hybridization with RNA Oligonucleotides. Dev. Dyn..

[32] Rodriguez A., Griffiths-Jones S., Ashurst J.L., Bradley A. (2004). Identification of mammalian microRNA host genes and transcription units. Genome Res..

[33] Zhang J., Choi E.H., Tworak A., Salom D., Leinonen H., Sander C.L. (2019). Photic generation of 11-cis-retinal in bovine retinal pigment epithelium. J. Biol. Chem..

[34] Voigt A.P., Whitmore S.S., Lessing N.D., DeLuca A.P., Tucker B.A., Stone E.M. (2020). Spectacle: an interactive resource for ocular single-cell RNA sequencing data analysis. Exp. Eye Res..

[35] Choi E.H., Suh S., Einstein D.E., Leinonen H., Dong Z., Rao S.R. (2021). An inducible Cre mouse for studying roles of the RPE in retinal physiology and disease. JCI Insight.

[36] Mattapallil M.J., Wawrousek E.F., Chan C.C., Zhao H., Roychoudhury J., Ferguson T.A. (2012). The Rd8 mutation of the Crb1 gene is present in vendor lines of C57BL/6N mice and embryonic stem cells, and confounds ocular induced mutant phenotypes. Invest. Ophthalmol. Vis. Sci..

[37] Redmond T.M., Weber C.H., Poliakov E., Yu S., Gentleman S. (2007). Effect of Leu/Met variation at residue 450 on isomerase activity and protein expression of RPE65 and its modulation by variation at other residues. Mol. Vis..

[38] Kohno H., Chen Y., Kevany B.M., Pearlman E., Miyagi M., Maeda T. (2013). Photoreceptor proteins initiate microglial activation via Toll-like receptor 4 in retinal degeneration mediated by all-trans-retinal. J. Biol. Chem..

[39] Lewandowski D., Foik A.T., Smidak R., Choi E.H., Zhang J., Hoang T. (2022). Inhibition of ceramide accumulation in AdipoR1-/- mice increases photoreceptor survival and improves vision. JCI Insight.

[40] Yu C., Roubeix C., Sennlaub F., Saban D.R. (2020). Microglia versus Monocytes: Distinct roles in degenerative diseases of the retina. Trends Neurosci..

[41] Kim J., Wu H.H., Lander A.D., Lyons K.M., Matzuk M.M., Calof A.L. (2005). GDF11 controls the timing of progenitor cell competence in developing retina. Science.

[42] Schallus T., Jaeckh C., Feher K., Palma A.S., Liu Y., Simpson J.C. (2008). Malectin: a novel carbohydrate-binding protein of the endoplasmic reticulum and a candidate player in the early steps of protein N-glycosylation. Mol. Biol. Cell.

[43] Galli C., Bernasconi R., Solda T., Calanca V., Molinari M. (2011). Malectin participates in a backup glycoprotein quality control pathway in the mammalian ER. PLoS One.

[44] Chen W., Yang S., Zhou Z., Zhao X., Zhong J., Reinach P.S. (2017). The long noncoding RNA landscape of the mouse. Eye Invest. Ophthalmol. Vis. Sci..

[45] Tworak A., Kolesnikov A.V., Hong J.D., Choi E.H., Luu J.C., Palczewska G. (2023). Rapid RGR-dependent visual pigment recycling is mediated by the RPE and specialized Muller glia. Cell Rep..

[46] Kim T., Croce C.M. (2023). MicroRNA: trends in clinical trials of cancer diagnosis and therapy strategies. Exp. Mol. Med..

[47] Krizaj D., Cordeiro S., Strauss O. (2023). Retinal TRP channels: cell-type-specific regulators of retinal homeostasis and multimodal integration. Prog. Retin. Eye Res..

[48] Brown R.L., Xiong W.H., Peters J.H., Tekmen-Clark M., Strycharska-Orczyk I., Reed B.T. (2015). TRPM3 expression in mouse retina. PLoS One.

[49] Hao Y., Hao S., Andersen-Nissen E., Mauck W.M., Zheng S., Butler A. (2021). Integrated analysis of multimodal single-cell data. Cell.

[50] Korsunsky I., Millard N., Fan J., Slowikowski K., Zhang F., Wei K. (2019). Fast, sensitive and accurate integration of single-cell data with Harmony. Nat. Methods.

[51] Finak G., McDavid A., Yajima M., Deng J., Gersuk V., Shalek A.K. (2015). MAST: a flexible statistical framework for assessing transcriptional changes and characterizing heterogeneity in single-cell RNA sequencing data. Genome Biol..

