# Supplementary material for: Conditional deletion of miR-204 and miR-211 in murine retinal pigment epithelium results in retinal degeneration
Source: J Biol Chem. 2024 May 4;300(6):107344. doi: 10.1016/j.jbc.2024.107344 (PMC11140208; doi:10.1016/j.jbc.2024.107344)
Supplement: Supplemental Figures S1–S3 and Tables S1–S3 [file mmc1.docx]

**Supplementary Information**

**Conditional deletion of miR-204 and miR-211 in murine retinal pigment epithelium results in retinal degeneration**

Samuel W. Du^1,2,*^, Ravikiran Komirisetty^3^, Dominik Lewandowski^1^, Elliot H. Choi^1^, Damian Panas^4,5^, Susie Suh^1^, Marcin Tabaka^4,5^, Roxana A. Radu^3^, Krzysztof Palczewski^1,2,6,7,*^


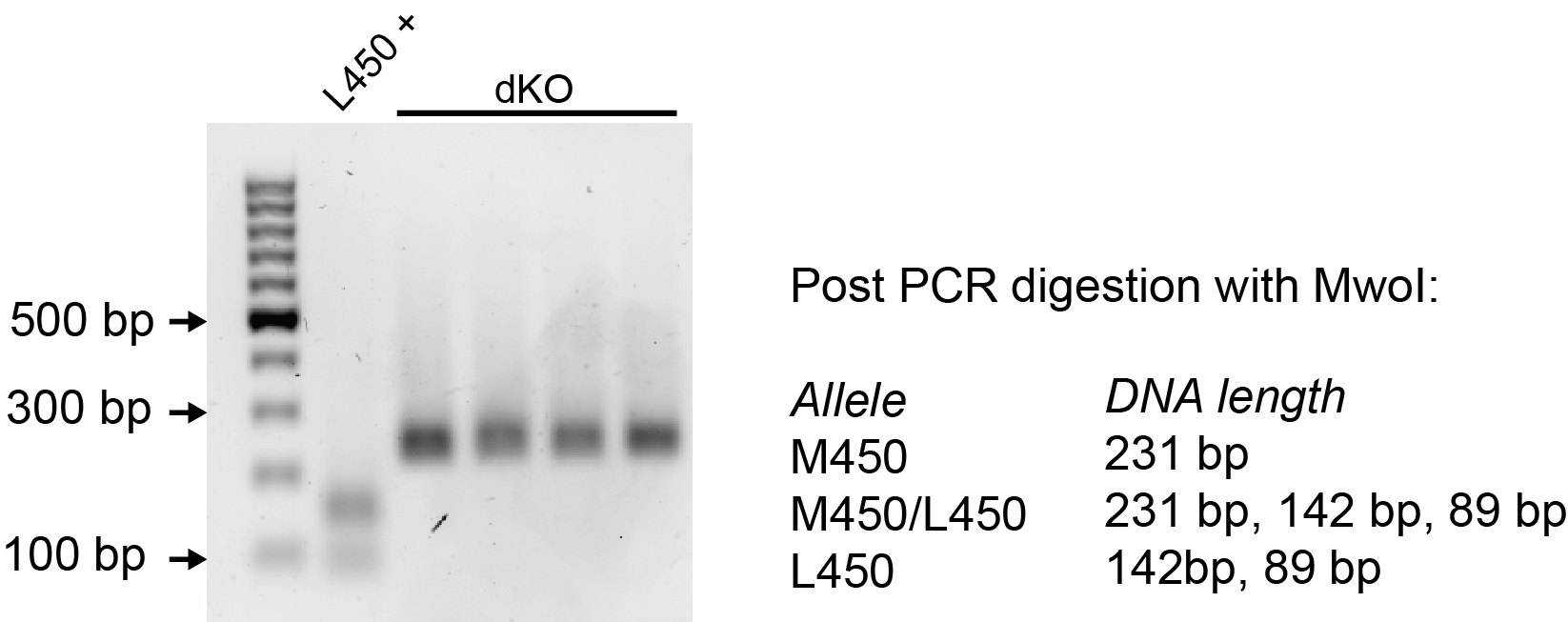


**Supplemental Figure S1. RPE65 L/M 450 determination.** After PCR of the RPE65 locus flanking the 450^th^ codon, restriction digest of the PCR product with MwoI cleaves the L450 allele (142 bp and 89 bp products), but not the M450 allele (231 bp undigested). In comparison with an RPE65-L450 mouse, dKO mice express RPE65-M450.


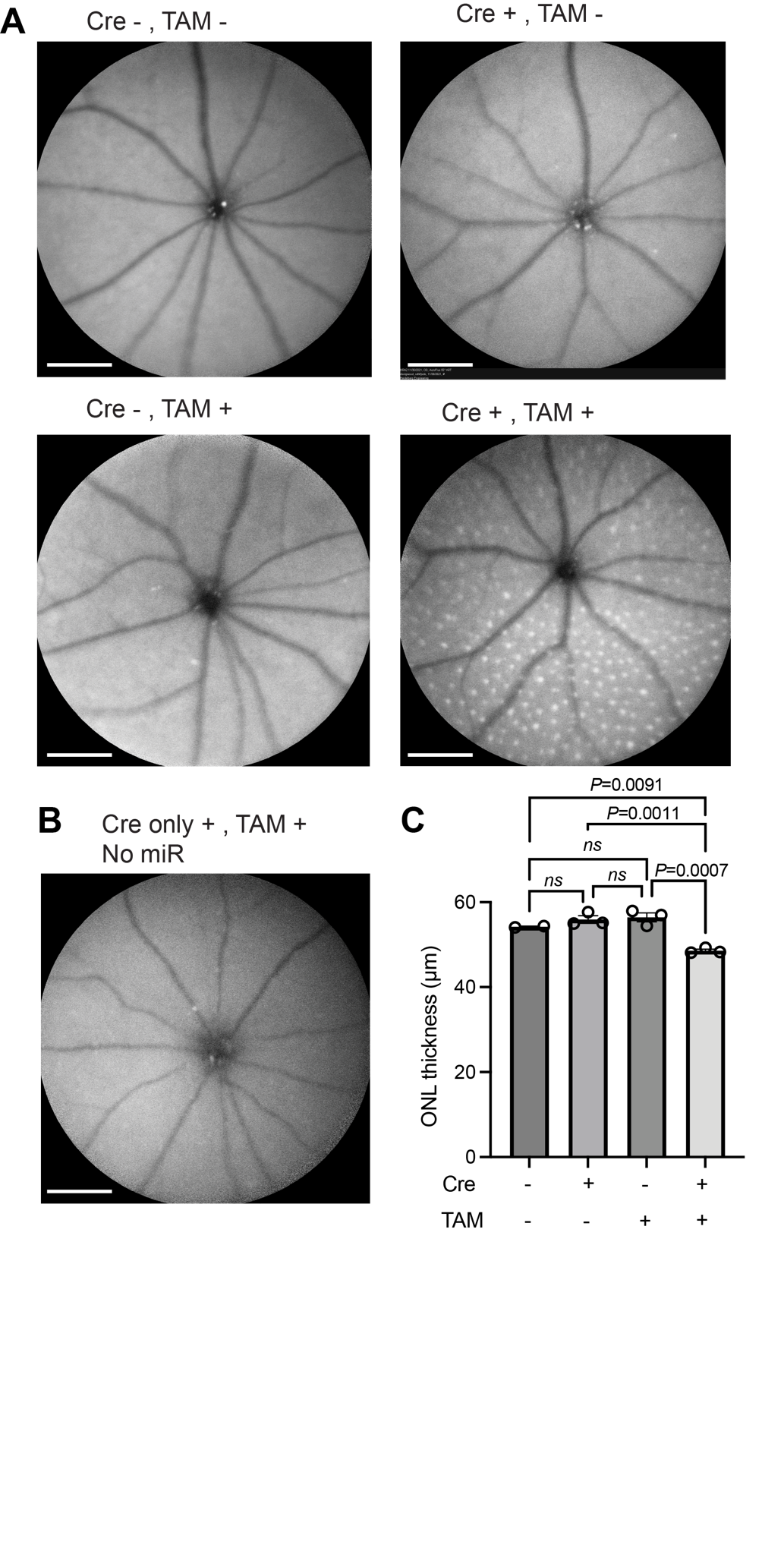


**Supplemental Figure S2. Assessment of control conditions for dKO mice.** **(A)** SLO fundoscopy at 4 months post-treatment with corn oil vehicle (upper) or tamoxifen (lower) in miR-204^fl^ x miR-211^fl^ mice, without RPE65-ERT2-Cre (left) or with RPE65-ERT2-Cre (right). Scale bar indicates 500 µm. (**B**) SLO fundoscopy of RPE65-ERT2-Cre mice without miR alleles, at 4 months post-induction. Scale bar indicates 500 µm. (**C**) Quantification of ONL thickness by OCT, 4 months after induction with tamoxifen (TAM); same mouse genotypes as described in (A), n ≥ 2 mice. Statistical significance calculated by one-way ANOVA with Tukey’s multiple comparison test.

**
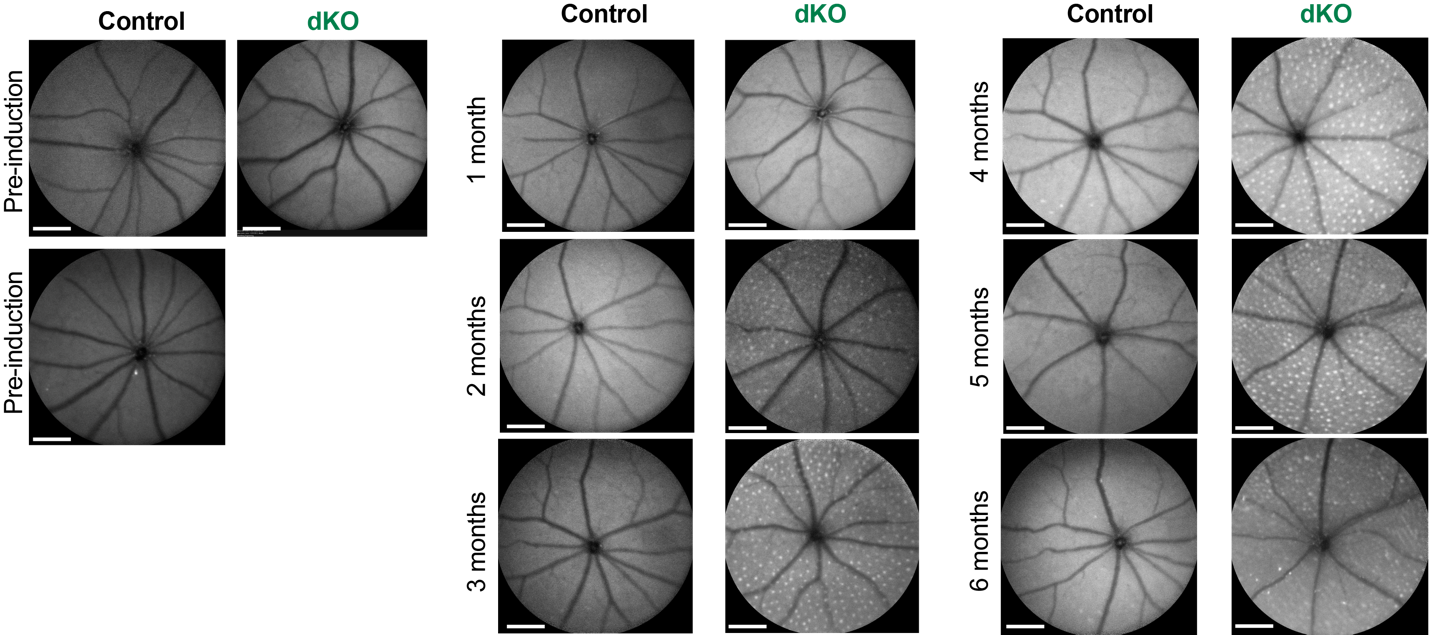
**

**Supplemental Figure S3. SLO fundoscopy changes in control *versus* dKO mice**. Autofluorescent foci were noted in dKO animals as soon as 2 months post-induction but were not observable in control animals. Scale bar indicates 500 µm.

**Supplemental Table S1. PCR primers and cycling conditions.^a^**

| *Gene* | *Primers (5’ to 3’)* | *Cycling parameters* | | *PCR bands (bp)* |
| --- | --- | --- | --- | --- |
|  |  | *Temp (°C)* | *Time* |  |
| RPE65-ERT2-Cre genotyping | **RPE65_E14f**:  CTTCCATGGACTGTTC  AAAAGATCC  **RPE65_E14r**:  AACTTCCAGGAGTAAG  TTCTGTCC  **RPE65_CreERT2**:  GCATAACCAGTGAAAC  AGCATTG | 94  \|94  35x \|63  \|72  72 | 5 min  45 sec  30 sec  1 min  5 min | 449 (Cre+/+)  449 + 146 (Cre+/-)  146 (Cre -/-, WT) |
| miR-204^fl^  genotyping | **NDEL1:**  ATTGAGCCCATAACTTT  CCTAAGACAAGGG  **NDEL2:**  ACCCCAGTAACTGCCC  ATGACTTTGG | 94  \|94  35x \|60  \|72  72 | 5 min  30 sec  30 sec  1 min  5 min | 438 (fl/fl)  438 and 308 (fl/WT)  308 (WT/WT) |
| miR-211^fl^  genotyping | **NDEL1:**  ATTTCTTATTCATGGCTT AGCCCAGTGGG  **NDEL2:**  TCCGATAAAATCTGCCAG CACGGG | 94  \|94  35x \|60  \|72  72 | 5 min  30 sec  30 sec  1 min  5 min | 555 (fl/fl)  555 and 426 (fl/WT)  426 (WT/WT) |
| *Crb1* WT allele (separate reaction from *rd8* allele) | **mCrb1mF1:** GTGAAGACAGCTACAG  TTCTGATC  **mCrb1mR:**  GCCCCATTTGCACACT  GATGAC | 94  \|94  35x \|65  \|72  72 | 5 min  30 sec  30 sec  30 sec  7 min | 220 |
| *Crb1 rd8* allele (separate reaction from WT allele) | **mCrb1mF2:**  GCCCCTGTTTGCATGG  AGGAAACTTGGAAGAC  AGCTACAGTTCTTCTG  **mCrb1mR:**  GCCCCATTTGCACACT  GATGAC | 94  \|94  35x \|65  \|72  72 | 5 min  30 sec  30 sec  30 sec  7 min | 244 |
| miR-204^fl^  recombination (Cre-loxP recombination) | **Lox1:**  GGAGGATTTATTCAA GTTCCAGA  **NDEL2:**  ACCCCAGTAACTGCCC  ATGACTTTGG | 98  \|98  35x \|61.2  \|72  72 | 5 min  30 sec  30 sec  60 sec  5 min | Before loxP recombination:  1200  After loxP recombination:  585 |
| miR-211^fl^  recombination  (Cre-loxP recombination) | **SC1:**  TCTTTGTGCACCACA ATCTAGAAC  **NDEL2:**  TCCGATAAAATCTGCCAGCACGGG | 98  \|98  35x \|64  \|72  72 | 5 min  30 sec  30 sec  60 sec  5 min | Before loxP recombination:  1739  After loxP recombination:  314 |
| RPE65 L/M 450 | **RPE65-LM450F:**  GCATACGGACTTGGGT  TGAATCAC  **RPE65-LM450R:**  GGTTGAGAAACAAAGA  TGGGTTCAG | 94  \|94  35x \|60  \|72  72 | 5 min  45 sec  30 sec  1 min  10 min | Digested with MwoI:  M450: 231  L450: 142, 89 |
| mm-miR-204-5p | Qiagen #MS00032557 (no sequence provided by Qiagen) |  |  |  |
| mm-miR-211-5p | Qiagen #MS00001897 (no sequence provided by Qiagen) |  |  |  |
| GAPDH (qRT-PCR) | **GAPDHF:**  CATCACTGCCACCCAG  AAGACTG  **GAPDHR:**  ATGCCAGTGAGCTTCC  CGTTCAG |  |  |  |

^a^PCR products were separated and analyzed on a 1% agarose gel in 1X TAE pre-stained with SYBR Safe (Thermo Fisher #S33102) and compared to the molecular markers (GeneRuler 100 bp, Thermo Fisher #SM0243; or GeneRuler 1 kb, Thermo Fisher #SM0313).

**Supplemental Table S2. Sequence of miR-204^fl^ targeted allele**

1. Outside 5' Arm (9825 bp): Plain text (e.g., ATCG)
2. Outside 3' Arm (13673 bp): Plain text (e.g., ATCG)
3. 5' Arm (5550 bp): Bold and italic text (e.g., ***ATCG***)
4. Distal LoxP (80 bp): Lightgray text, highlighted in yellow (e.g., ATCG)
5. Middle Arm (MA) (525 bp): Green text (e.g., ATCG)
6. Neo Cassette (2815 bp): Red text (e.g., ATCG)
7. 3' Arm (2100 bp): Underlined text (e.g., ATCG)
8. LoxP: Red text, highlighted in yellow (e.g., ATCG)
9. FRT: Underlined in red (e.g., ATCG)
10. Exon: Highlighted in pink (e.g., ATCG)
11. Probe: Highlighted in sandy brown (e.g., ATCG)
12. Oligo (forward): Highlighted in lime (e.g., ATCG)
13. Oligo (reverse): Highlighted in magenta (e.g., ATCG)

ACCACAGAATGCTGTTTACTGTCTTGTTCCCTCTGATTTGCTCAACTACTTTTCTTATTCTGCCCAGGGGCCCCCTGGCTAGTGATGGCATGAGTGGTGCCCTCCTGAATCATCTAGCAATTAAGAAAATAGATAGCCCTGCAAGCCTATACCATTGACACAACTCCCAGCTGAGGCTCCCACTTCCCAGGTGACCCTAATTTGTGTCAACCTAAAAGTAGCCAGAATGTACTAATTTTAAAGTTTTAGCTATTTTTCTGGAAAAAAAAAAAAGATATGTGTTTTTGTGTTTTCTACCGATTATCATGATAATTGAAATAAAGGTGGCCTGCATCCCATGCCCCACAGGTCATGTTCTCCTGATATATCTTGTTACATCTTATAACCATTTGTCTTGGAACCCAGGCCCTTCTGCTGTAACAGTGACCCAAAGCAAAGCTACATACAAGGGATCAGAGATGACTTCCTACTTATAAGTCATCCCTAATACAGATTCAAATGTAATGAGAGCACCGTGGTATCAAGACTCAAAACCCTTCTTTGGTTTGTTCTACTTACATAGTTCTCATCAACATGTGTATTTTTGTCCAGAGTAAATCAGAAACAAAGAACAACTCAGGATTGTAAACAAGCCTCGCCATACCCAACTGAACCCTAAACTTTAGAGGCTTAACAAGAAAGTTACACCATTTGGTTTATATTTTATATTTGTCTTGTGTCCGACACTAACTATGGAGCTCAGTCTTAATCCAGGAAATAGGCAGCAAGCTAAAATGATATCACTAGGAAGTACAGGAAAACTATTGGAAGACAGATGGATGTTTTGCTGCATCTTTTAAATCTAACATTCCAAGTAACTACTGAAGATTAGCTGATTGGCCTATGGAGAGAGCCTGTCCGGCTTCCATAAATAACATGTGTCTGGCTTACCCAAGATGCTCACTGGGGAAATTACCCTGAATAGCACCTGTAACATTGGAATCAGGCTTGCTCTTTCCTGTTTCACCTTTGAGCTTTTAAGAATCATTGATACAAATCATACTGAGACTAAGCTGTCCTAGGACATTTAGACTACACCTCAACCCTGATGCCGTTGACCTCTTAAACTGAATAATCCAACAGAATAGCCTCCTCGTTTCCTGTGCAATGCATAGTGTGTTGTTACACATTATTAGGTAACATTTTCAATCACAGGAGAGTAAAAATAGAAGGGCAGAAGCCATGCTTCCATCCAGAGCATCATACTCATAATGGGAACAACTCAAAGGTAAATCCCAGGAAGCAATCCTCAGAGCACGTGGCTAGGAGCCTGGCAGCAGGCAGTGGGAAAACAGAAAGGCGTTGGCTCTCTTGTTAGAAGGAAATTATCTGAGAGAAGGAGAGAAGCCCATTCTCTGACTCAGTGGAGGGAATCTTTGAGGATACTGAAGAGAAAGTGGCCTAAAACCTCATGTCAGTTATCCAATCTGGCTTTCCTTCACTTGAATAAACCTGAAGAGCTAACTAGTGATTATGCAGGATGAAGCCATTAAGGTAGGATGGAGCTGTCCGAGGACTAATAAGCTTATGAATGAGTATGTCTGATAATGACAGTTATAAAAAAATCTATGAATATGATTAATCTTCGGAGCTAGTTCACTGCGTTCCTTGTTGAGAGATCAATATACTTGTCATCATCTATTCATCCCTAGACTTTCCTTTGATTTCTAACTTCGTTATTCAGTCCAAGCTAAGTGTTAGGTCTACGTGTAGATGCGGCTTCTGCTCTAACTTAGTGGGTGGACAAATTCAACGTGTTGTATCACCTTACACACTTAACGAAGCTACAGCAAGCACTCTGAAAATAGACCTTCCCTTTCTCTCTTATACTCCGTTGGTCACAACAATTTTTTTGTCTCACTCCTTTCTCATTATCATTTTATAAAAATAACTTGAGTATTTAGTATCAAAAATGCTTGAGAGCCTGTGTTATAGATTTTGGCAATTTGAGACTACCTGATTTTCAGATTAGGAATGTTTTGCCAACACCAAAATCATAACTATTGTGACTTTCTCATTCACACAACACAGGTTTCTACAGAAAACAAATGTTCATAAATCTCATTAGAAAAAAATAAATAATTTCAAAAGACGGTCAAATGAATAATATATTCACAACTATGACAC***CTAATTTCCATAATATGCAAAGTATTTATAAATCAAAAAGAAAAATGTGTAAAACCCTATTGAAAATGGAGAAAAGCATATCCATAGGAAGATTAGGGGGAAGAAAATAAAGATTTAACATCATTCCACAATGAAAGAATTTCATCAATCAAATTGGAAAAACTCAAAGATCAGGATAATCTAATGTCAATAAATGTCACATAAATATGAACTGGTAACATCTTTGTTATATGTGTAATGTTAAATAATGTGCAAACCTTATTTAGCAAATACTTTAGAACCCCCTTCAAATGTATCAACCCTTTGAGTCTATATTTTCAATATCTGGAATTTACTCTGTATATATCTGCTATAATATTGTTCACAGTAACAAAACAGAAATAACCTAACTATTATTTGATAATTTAAAAACTATACAGAGACATGGAGTGAAATACTAAAATCATTGTAGATAATGAAATAGTTCCATTTGGGATCATATAGAAAACTAGCTAAAGGTATCTTTTAAGTCAAAAGTCTGTTCTGTAATGTAATTGATTGCCTGACATTTTATACTCATATGCTTATAAAATACCCTTAAGTATTCTAGTGGATAAATATTATTGACTGCGAGCCATATGAGAGAGAACGGCAGGAAGTGACACCTACACGGTATACTTTTAAGATTTCTTGGTGTTTTGTCTCATTTAGGGCTTTTATTTCTATGATGAAAAATCATGAACAAAGCTACTTGGAGAGGAAAGTGTTTATTTGTCTTACATTTCCATGTCAATTGAAGGAAGTCAGGACAGGGACCAAACAAGGCAGGGACCCAGAAGCAGAAGCTGATGCAGTAGCCATGGAGGGGTGCTGCTTGCTGACTTGCTCCTCATGGCTTGCTCAGCTTGCTTTCTTATAGAACCCAGGACCATCAGCCCAGAGATAGCACCACTCACAATGTGCTGAACCCTCCTCCATCAATCACTAATTAAGAAAATAAATATCTTAAGCTGGATCTTTTAGAGGCATTTTTCTCAATTGAGGTTCTCTCCTTTCAGATGATTCTAGCTTATGTCAAGTAGACATAAAACTAACCAGCCCAGTGTTGACTTTTACAGTAAACAAAGATATTTTTCTCTCATAGAGGATGTGGATAAGCCTTTGGAAGTAATCCCCCCATCCCTCATTCCTGTAACACAGAAGTTTGAAACCTTTCTTCCTGCACACATACATAGCATGCTTTAAAATACTTACTATGTAAAATTAAGATGTACAAAATAGAATTGCAAGACACATCCAGTAAAACCATCTCACATAGTTCCCCATCCCCTCCCCTGGATGAGTAGCCACAACCTACTCATTTGGCAAAACTAACTACAGCACACCACTCTCTACTGTAGTTCTCATGTGGTGTGCTTTAAATTAAATTTTAGATTGTTCACCCAACTGTTGGATCTTTTAACCCTCATTTCCTTGCTCCCCTACCCCAGCTCTGAGCTGACTCGCATCCTTCCTAAGCAGTGACTACAAACCTGTCTCTCACTGCATTCAAAATAGCTCTTCCATGGTCCATGCATTCTTGGCTCATTCTTCAGAATGAAGAATCATGAGAGTCCTATCAGGAAACTTAGGGGGGAAAGAGGCTGGTTAGAAACACATGAGCAATGTTTTCAAGTCATGTGTGGAGGTTATATAAAAATGATGGGCCAATATAACAGCTTTCAAGCTTTGATTTAATAATGGCTAAAATCAGAACACAAGTCATGGTCCCTCTTCAGAGATGGCAGATTACAACATAGCTTACACACGGTCCCAGTACTAAACAATAAATAGATGTCCTGTTGAAGTAGGGAGACTAGAAACACCCCACATCTGTGTCCCTTCCAAGAGGAGACTATGTTACTGACAACTAGTCATTTCTCTTTCATCACAGTCTAGATACCACAAAGTCCAGTTCCCTTTTCTCTATTAAGTTGTAACTCATTCAACAATGGTTTCATGCTGAATGTTGTCCATATGGTCACTGTAGCAAAGTTTCTCCAATATACTCTTTATCTTACTACAATTCTAAGATGTTTCCTATGTGTGTGTGTGTATATATAATTTCTTCAGCTTTTTAAAATGTAGCAAAACCTGTCATTTAAAATTAATAGCGATTACATCGCTCCTTTTCCCATGGCATTGCCTCTTTGGAGCTCCTTCTTTACAAACATTTGCCTGAGTGCCTATACTATAGAAAGCACTGTAGAGGTTGCATAGTACATAAGGTGTAGCTTTGTGCTTGAAGAACTTAGTCTTTGAAGAAGTCAACAAAGCTCGTGTTTATCGAGTGCTATTCTAAATCATTTACAAGGAACAGCTGATTTAATCCCCACAACACCCATTCTTCATCTACTCCTTAGTAAGCAAGCTGAACCACAAAGAGGCCAAGGAATGGCCAAGGTCAGACTGAGACTAAATGGCAGATCCAGGAAATCTGTGTCTGAGCCCACGCCTGTACAAGCCTGCTCTAAGGGCTGGCTGTGTGTACAGAGGCTGCACTATAGAGTAGCAAGTGTTCTGTGTCCTTTGTGATACAAAGTACAGTGGCAATTCAGACAAAACATTTGGGGGTAGAATATTCATGGGGATGCCTGTCAAGCAAAATTTGACAGGTATAATGGGGGGCAATCCACTTACATGCCACCATGTAGGTGAAAGCTCTCAATAGGCAGAATGTTAGTGCAGTGCATAGAGTCGACGATTGCATCATTTCCGTTCCTTTTTATCTTCAAATGGCTCAGGACAACTTTCCTCTGCAAACAAGAATGGGAAGATGTGAACTCAAGTTAGTTTTTATAAAACATTATAATAATCAAATTCATAAGGAGGGGCATGTATGTTTCGTTGCTTCTTTTATTAACAGAGAGTCTTAGGGAGAAACTAAACTAGAATATAGAAACAGAACATACATAATACAGTGTGGCTTATATGTGTGCCCAGACATCCAGATGATCGGTAGATCTCATCTAACTGCTCTCATTCTCTATTTAGAAATGCCACTCGGAGAAAGCCAAATGTTCTACCTAAATTCACAAGGCATTGAATTGAATTCTATCTGAACCTAAACTACACACAGCCTGTCGTTCCCTGCATGGCAGTGTCTTAGCTTGAGGATGGTTCTCTGTTTGGTACAATGCCCTAAGAATTCAGAGACAAAACAAACAAACAGTAAACAAGAATAAAAAAAAAACTCCCCTAGACACCTCAACAGTGAGCACCAGCATAGCAGCATCAACCTTGGTTTGGTACAAAGTCTTTAGTCCTTTATCCTGTACCTCCTCACTCCTCATCTTCTCTACTGTGTCCTGGGGACACTAAAGGATACAGCAAGACAATCAGGGACACAACCAATGTACCCCTTTGTCTACTTTTTAACTGTACCACCATGATTTGCCTCCTTATTTATATGGGATCCTTTGAGATTTTTCTTCCTGAGGATGTTATATACCTAGGGAAGAATAAACTGAAATGCAAGCATGTGTGGTTCATTCTGCAGGAGAGATGTAACAAAGCCAGATTTATTCAACTTTATTATTTATTTGTTTATTGCTCAGTTCCTTACCCCAATACAAGAAATATTGGGGCTTGTATGGTTGCTACCTATTAGAAGCTTTGCTTACAGTATTTAGCAAGTGGTCTGATTGCCACACACAGAGTATATTCTTATGAGCTTAATTGTCCTCAGAAGTAAACTAATTATGGACACTCTGGGAAATCTACTGTCACTACTCTTGGATTGTTACTTTATGAACAATAGAGAAGCCAAAGAAAGTGACCCCATGTACTGCAGGATGCTGCATGTCCGGTGCTTTGAGGGCTAGTCCTCCATGGTTCTCTCAACGCACTCACTGTTTCAACAACGAACCTTTATCATCTCATAGGCCTGAGGAAGGGCTCAGTGGTGACCCATGAAAACGGGCATAATGCTTCTCACCAGCCATCTGCCCTTGCTCAGTCTGTGGTCCCAACAAATGCCACTAACTGGGGCTTCTCTAGAAATCTAGCTCAGTCCTAAAAAGCTGGGGTCATAAGTTTCACCATGAGGGTCTGCAGTTCGACTAAATTCTGTTCTGATGCTGCAAAGACCACCAGGAAGGCAAGTGCAGACTCGTTTCCTTTGGCTCAGATTGAGTAGCAGGTGTTTTTTGCACCAGCTAGACAAAAGCTGGCTTGGTTCCCCAGTGAGCAGCTCCCAGACAGGGTCTTACCTGTGAATGTCTTTCCCGTGAGGGAGGCAGCAAAACCAGCTAGAGGGGAAATCTAACACACTGCAATAGCTTTGATGACTGTTAAAGAAAAAGAGAGGGAGTGTTGGGGAGACAGATACCCCTCTGGCTAGCCAATGAGCGAGCGAGAAAAGAGCAGAATGAAAATCTTAAGTAAATATCATTGTTATTGTTTAGTAGCAAATCAAATAGGTTATCGTGTAACCAGCTAGTATTTTAATTCACACGTAGCTAGTCATTTAACAATGAAAGGTATGTGTTTCAAACAAACAAAGAAACAAACAAAAGTCGCAGAAACGTTTCTAAAAAGAAGACAGGCATGAAGTTGGTTCTTGTTGTCCTGGAAGACACCAAGCTGAAAGAATTGTAGTTATTCTATGTAACAGGCCAGCAAGCACGGCTTCTGGGTTTTACCAGTGCTAAGCACTGGAACAAGCACTCAGTCCCTTCATCTCCCATATCTGTCTTAGATTAAAACATAATAAAATATGTTTCCTTCAAGAGAAGGGATGTTGATCTTATGAATCTGCTTGTGTTTGGTTTTGAGAAGTCTGTGTGATTCTTTGCACAGCTCTATGTGTGCAGTATGGGTTCAGTCACACACAGAGACTTATACAAATTGGGTGGTCCATCTGCTTTAATAAAATGTGTATCCGACCATTGGGTTGTAGAAATTTCAAAGCTTTTGTTTTTTGACAGGATGCCTGCCGTTTTTCTCTCTTGACTCCCGCTTGTTTTATTCATTTTGGGGTAGTTGCCAATTAGACCCAATTGGTAAGTATAATTAACAAAAATCTTGAATTTCCTCTTAAACCCCAACCAAGAAAATTAACCATAGATGCTATAATTTCCTTTAAAAAATTCCTAACCACATCACTCTTCTGACTGCTGCTTATCTCTTCTGAGACATCCATTCCCATCAGACTTTATTGATTCCTTTTTACCTGTTACCCTGGAGATGCCACCTAGGACAGGATAGCAGCATCCTAAATATGACTAGAAGAAAGGAGGATTTATTCAAGTTCCAGATACTATGTAAGGTATCACCATATAAACTGTCTCTTTTAATACTTGACAAATTCATTCAAGGTAGAAATTGCATAGCTTTATTGTAGTTATAAAATGATATCTCAGATAAGTTATGCCTTTAATATTATGTCCACCTACTTAGGCACCTGAGTACATACCTAGTCAGATGTAACTCCCAAACCTTTACTACCCCTTCCATCCACGTACAAAGCATTAGCTCCCTTAAGAGAAGACTCGCTAAAACTTGGCTACCTTTGAAAGCTTATGGGCATTG***ACTAGTTGGCCATGTACATATCACGCGTATAACTTCGTATAGCATACATTATACGAAGTTATGCCACTAGAGGATCCCCGCTTCATTCAGCACCTAGTTGAGGACTCACTCTGTTCAGGACTTGGCTAAGCATTGCTTTGAACAAAATATCAAACAAGGACTCAAGGGGCAGAGAATGCTGGTCAGTGGCTAAGATGCCGGAGAATCAAGATGAGCAGGAAATGAAGAGGTTGGCTAAGAGGGGCAGAGGAGGCAGGCGGAGGAGCTCCTGACCGTGTACCATGGCTACAGTCCTTCTTCATGTGACTCGTGGACTTCCCTTTGTCATCCTATGCCTGAGAATATATGAAGGAGGCTGGGAAGGCAAAGGGACGTTCAATTGTCATCACTGGCATCTTTTTTGATCATTACACCATCATCAAAAGCATTTGGATAACCATAACATGAAAATTACCATCATTGAGCCCATAACTTTCCTAAGACAAGGGTGACAATTTGAAACATCAAAGAACCTTACCCAGGGAATTCAAGAAGTGAGAAGAGTGAATCAGATTCTCCCAGATTAACAACCCCATGTTCAAATTCTAACTGGTAAAGGAAACTGACGTACGTTCGTGGGATTGTGTCCGTGTCGCGAAGTTCCTATACTTTCTAGAGAATAGGAACTTCCCGCGGATAACTTCGTATAGCATACATTATACGAAGTTATGTAGATCCAGATCTAGAGTAAGACAGAAGTCTGGAAGACATGGGAGTGAGTTGTCAGGTGATCCAGGAAGAGACCTTCTGCAATCCAGTGACCAATTAATTACAGCAGAAAGGACCATCGGGAAGGAAAGCCATACTCTCCAGGAACGTCATTAGTCGGGATCTTCAGTTGCTACAAGAAGCAGATGTCAAACGGCCTTCCCCTAACCATGTGAGAAGTGAGCTTTCACTGGCCCGGGTGTGAAGTGATTCTAATGGAATAAATGGATTTGCTAAGGAATAGTTTCCTCAGAAGAAATCCTGGGAGCAAGTGGGGAAAGCTGACTCAGCAAAACAGAGCTGTTTCTTGAGGACGATGCCAATAGCAATCATTTGACCAAACTGAAGTGGCCGTCAGGAGGCATGAGGATCTGATATCAGGGAGCTCTCAGACGTCGCTTGGTCGGTCTTTATTCGAACCCCAGAGTCCCGCTCAGAAGAACTCGTCAAGAAGGCGATAGAAGGCGATGCGCTGCGAATCGGGAGCGGCGATACCGTAAAGCACGAGGAAGCGGTCAGCCCATTCGCCGCCAAGCTCTTCAGCAATATCACGGGTAGCCAACGCTATGTCCTGATAGCGGTCCGCCACACCCAGCCGGCCACAGTCGATGAATCCAGAAAAGCGGCCATTTTCCACCATGATATTCGGCAAGCAGGCATCGCCATGGGTCACGACGAGATCCTCGCCGTCGGGCATGCGCGCCTTGAGCCTGGCGAACAGTTCGGCTGGCGCGAGCCCCTGATGCTCTTCGTCCAAATCATCCTGATCGACAAGACCGGCTTCCATCCGTGTCCGTGCTCGCTCGATGCGATGTTTCGCTTGGTGGTCGAATGGGCAGGTAGCCGGATCAAGCGTATGCAGCCGCCGCATTGCATCAGCCATGATGGATACTTTCTCGGCCGGAGCAAGATGAGATGACAGGAGATCCTGCCCCGGCACTTCGCCCAATAGCAGCCAGTCCCTTCCCGCTTCCGTGACAACGTCGAGCACAGCTGCGCAAGGAACGCCCGTCGTGGCCAGCCACGATAGCCGCGCTGCCTCGTCCTGCAATTCATTCAAGGCACCGGATAGGTCGGTCTTGACAAAAAGAACCGGGCGCCCCTGCGCTGACAGCCGGAACACGGCGGCATCAGAGCAGCCGATCGTCTGTTGTGCCCAGTCATAGCCGAATAGCCTCTCCACCCAAGCGGCCGGAGAACCTGCGTGCAATCCATCTTGTTCAATGGCCGATCCCATGGTTTAGTTCCTCACCTTGTCGTATTATACTATGCCGATATACTATGCCGATGATTAATTGTCAACACGTCTAACAAAAAAGCCAAAAACGGCCAGAATTTAGCGGACAATTTACTAGTCTAACACTGAAAATTACATATTGACCCAAATGATTACATTTCAAAAGGTGCCTAAAAAACTTCACAAAACACACTCGCCAACCCCGAGCGCATAGTTCAAAACCGGAGCTTCAGCTACTTAAGAAGATACGTACATAAAACCGACCAAAGAAACTGACGCCTCACTTATCCCTCCCCTCACCCGAGGTCCGGCGCCTGTCGATTCAGGAGAGCCTACCCTAGGCCCGAACCCTGCGTCCTGCGACGGAGAAAAGCCTACCGCACACCTACCGGCAGGTGGCCCCACCCTGCATTATAAGCCAACAGAACGGGTGACGTCACGACACGACGAGGGCGCGCGCTCCCAAAGTTACGGGTGCACTGCCCAACGGCACCGCCATAACTGCCGCCCCCGCAACAGACGACAAACCGAGTTCTCCAGTCAGTGACAAACTTCACGTCAGGGTCCCCAGATGGTGCCCCAGCCCATCTCACCCGAATAAGAGCTTTCCCGCATTAGCGAAGGCCTCAAGACCTTGGGTTCTTGCCGCCCACCATGCCCCCCACCTTGTTTCAACGACCTCACAGCCCGCCTCACAAGCGTCTTCCATTCAAGACTCGGGAACAGCCGCCATTTTGCTGCGCTCCCCCCAACCCCCAGTTCAGGGCAACCTTGCTCGCGGACCCAGACTACAGCCCTTGGCGGTCTCTCCACACGCTTCCGTCCCACCGAGCGGCCCGGCGGCCACGAAAGCCCCGGCCAGCCCAGCAGCCCGCTACTCACCAAGTGACGATCACAGCGATCCACAAACAAGAACTGCGACCCAAATCCCGGCTGCGACGGAACTAGCTGTGCCACACCCGGCGCGTCCTTATATAATCATCGGCGTTCACCGCCCCACGGAGATCCCTCCGCAGAATCGCCGAGAAGGGACTACTTTTCCTCGCCTGTTCCGCTCTCTGGAAAGAAAACCAGTGCCCTAGAGTCACCCAAGTCCCGTCCTAAAATGTCCTTCTGCTGATACTGGGGTTCTAAGGCCGAGTCTTATGAGCAGCGGGCCGCTGTCCTGAGCGTCCGGGCGGAAGGATCAGGACGCTCGCTGCGCCCTTCGTCTGACGTGGCAGCGCTCGCCGTGAGGAGGGGGGCGCCCGCGGGAGGCGCCAAAACCCGGCGCGGAGGCCGCATGCTCCAGACTGCCTTGGGAAAAGCGCCTCCCCTACCCGGTAGAATGAAGTTCCTATACTTTCTAGAGAATAGGAACTTCGTTCGAACATAACTTCGTATAGCATACATTATACGAAGTTATGGTACCGAGCTCGAATTCATCGTACGGGCCCGCTTCCTCTGAACAGGTTGTAATGTATATCAAGAACATCCTACTACCTTCTGTGGATGCTCTAAACTGTTTAGCCCAATCATACAGACATTCTTCTGTCTCAACCAAAGACCAACACTGAGTGCAATCATAAATACCAAAGTCATGGGCAGTTACTGGGGTATGTGATGACTATGTCATTTTTCAAATGCTCTCAACTGCATTGAATAACTGTTCAAATTATTCAAATTTTCCTCTGTCTTTTAGATGGCCTTCCATCTAAAGCAGCAGCTCTCAACCTGTGTGTCATGACCCCTTTGGCTAATGTTGTCTCCAAAAATATTTACATTATGATTAATAGCAGAAGTAAAATTACAGTTATGAAGTAACAATGAAAATAATTTTATAGTTGGGGGTCACCACAACATGAGGAACTGAATTAAAGGGTCACAGCATTAGGAAGGTTGAGAACCACTGCTCTAAAGAAAGATTTGTATTGAAAAAAATCTTAGCTCTCTATTTGGTGAAATGACTTGGCACAAGTTGTTTGTGTGTGTGTAAAACATTAGCCATCCTTGTTAATTAAAACACACTTTGAAAATCAAGACAATGAAGTCAAAACAACTTTGGGGAATAGAAGAAAATTTAAGGAAAATGGTTTCTTGGTGTTGGCCTGTCTTGTTCATCCAACCCTTGTCCAATCAAGCCAGGATCCCTCTCTTTTTGAGAAAAGGACCTTTTCACTTTGGAATTACTTATTCAGGGATGGGGCAGTTCTTTTAAGCAAGTCTGACTAGGTCATCTGCCTGGCATTCAAATATGATCTCCTAGATACTGCTCCATGGAGTTAAGAACTGTGAAAATTCTTTGCAGATTAGAAAGACTTAGGTTGGAAATAATTTAGACTTTTGTCCTCAAGATCAGTAAAACGTCCTGCTGGTGGTCCGTTAAAGTAGATTCTTTTCAGGGTAACAGGACTTTTCCAGTGGGACTTGGTTCCCTTCTTGTTTCCTTGCTAAATGAAAGACCCTCTAAAGTATTCCCATGTCTTCTCAAGCTTCTTTTACATACAATGCCATATGGATCTTCTGATTTACTCATTCCAAGATACTTACAGTTTCCTTGATTTAATATACCCTATTGTGTATGTTTCGTCTCTTTTTAATACATGCTTGATTTCTGCCTCTCTTATTACTAATGGCAACGGTCTTTTAATCTCTCAGTCTAAGAATCCACCTCCATCCAGGCATTTCCCTCCCTTGATGAACCTCATAGGCCATGTCCCTCACATCTTCCCCATCCCTACTTTACTGTCTAAAATTCTGTGGTAGCACTTCTTATTCATTAGTGATTAGTGTGTCCCTTTCTCTGCTTAGTTGCTGATCACCTGCCAGCTATGAACCTATAGAAAACAGAGCCTGTGTCTTAACCATTTTTATCAGCCTGAGAACTTAGCACAGTCCCTGGCACATGCAAATAGGTGTCTGATAGATCACTGCTGCCAATGGCATTACTTAGTTTTCCTAGACTTATTTTCAGCTTTGATTTCTAGTTTGACATTGAAGTTTAGTAGAAGAGCTTAGATGATATAGTCTAGAAAGGAAAGGCAAATGAAGTAACAACATCATAGATTCAAAGGAGAAAACCGAATGGGAAAGGGTTTGGGCAAAGATAACAGTGAGTGCCATGCCTGGTGCAGAAGGACATTCAGCCTGGAAAATCTGAGTGTGCTCTTCTTCCACTGTTTATTATTGTTATTGTGATTATTATCAAACCATTTCCAACTAAATCAATGCATTTAGAAGATCTAAAAGAAATATTTTTGCATTAGCCAAGTCTATCATGACTTCAGTATTTCCATTTATACATAAAAGGAACTATCTATTGACTTCTCCCTACTTACTGAAAGTAATAGTCACCCGTGACTTCTTTTCACTCTAGATCAGGATCAATGTATTTGGTAGCAACGCATAGCAAACCTTAGAAAGTCAGGCGAACAGAAAACAGATGGAAAAAGTTTGTGCTGAGTGGGTGCTTTTCTGCCATAATCATCAACTCACAGTTAGAAGAGATGGTATTTCATTTGCGAAGGGTCTCAAAGAATTGTGGATTAAGACTCTCATTTGCTTAATATGGCTAGATCTGAGGAGTTCACTACAGGGCCCTCCAAAGTGCCTCTGAGGATTGCAAACAATTTGTTTAGTCCCTTCAAAATGTTATTTGGAGCATTTCAGTCTCTTGAAACAAATGGATGTAAACGCAAAATGTAATTCATACAATTCTAAAGAATAACTAGAAATGTCACTAATGACACTAATGATTGTTTTAACAATAAAAGACATTCTAAGCCTAAACGCTCAACTCATCAGCCCCTAAAACAAAATGAGGAAAACAAGAGATTTCATGCCTTCATAGTGGCCAAATAGGCAAGCAGCAGATTTTTCCTTTCTCTTTTGGCACCCTCAACAAACAGGCTGTCCCTGTTCCACACTGGAAGCCAGGTTTCCAAACTCAGCAAAGGTCTACATGCCGCAGCCCAGAAGGCAAACACTCCAGTCCCCAAACGCAGGCAGGGGAATGCTCGACCCTAACAAGCCTTGGCATCTCTGGGTCCACGTGGGGACGTGTGGGCAATTCCTGCTGGAAAGACGCAGAGTGGAAAAAAAAAAAAAAAAAAAAAAAGAATAGGTGTGTGCCTTTGCCAAATCAGGGCGGGTGAAGATTGAAGCGTCAGCTTTCCTGGTCATCAAACAAATCCGAGCTGAGCCCTGCCTCATTAGCAGCATCTCGTCTACCCAGAGGGAGAGCACAGGAAGTGTGCACCGTCTGTGCTGCACATTAGGAATTGTTATGAAATCCTGGGGGAGGCTGGGAAGCAAGCCACAACTGAGCACAGCATGCCTGCGTGAAGTCTAGGCAGCCGTTCCCTGCACCTTGCTTTCCAGCCTCAGACCCTGGCCTGTTGAAACACAACAGCATGTGTTTTGCCCTGTTGATTTATATGTTCCCATTGGTCAAACACTAAGAACTATTTCACTAGACGTTGTTGTTCATAAACTTAGATACATAGGAAATGTCGCCTAATGACACTATCAAATAGACTCTAACCTCCCTTCATATCATGTTTTCAGTTCAAACTTGCTATAAATATTAGTCCCGCACCCTCCCCCAACCCTGTGCTAGTTAGTTTTCTATCACTGTAAAAAGCTTTTGAGCTTACATTAGTAAGTCGATTCTTTACTTACAAGCAAACTACCAAAGTAGAAAACATGGGTATAAATGAGACGTTTTCACTAGTGAAACTCCCATTGCGAGTTTCCTATACAGTCCCTAGTAGCTAGGTCCTGGGCTTGACTTGCTAGGGAGTTCATCTGCC

**Supplemental Table S3. Sequence of miR-211^fl^ targeted allele**

1. Outside 5' Arm (9825 bp): Plain text (e.g., ATCG)
2. Outside 3' Arm (13673 bp): Plain text (e.g., ATCG)
3. 5' Arm (5550 bp): Bold and italic text (e.g., ***ATCG***)
4. Distal LoxP (80 bp): Lightgray text, highlighted in yellow (e.g., ATCG)
5. Middle Arm (MA) (525 bp): Green text (e.g., ATCG)
6. Neo Cassette (2815 bp): Red text (e.g., ATCG)
7. 3' Arm (2100 bp): Underlined text (e.g., ATCG)
8. LoxP: Red text, highlighted in yellow (e.g., ATCG)
9. FRT: Underlined in red (e.g., ATCG)
10. Exon: Highlighted in pink (e.g., ATCG)
11. Probe: Highlighted in sandy brown (e.g., ATCG)
12. Oligo (forward): Highlighted in lime (e.g., ATCG)
13. Oligo (reverse): Highlighted in magenta (e.g., ATCG)
14. miR-211 (106bp) **CTGCTTGGACCTGTGACCTGTGGGCTTCCCTTTGTCATCCTTTGCCTAGG**

ATATATATATATATATATATATGTATATATATATATATATATATATATATCATGCACATTCCACACAATGCACATACACACACACACACACACACACACACACACTATCCATATATCCCCATTACCACACCCCAGAAACCATAATGCATATACCTATGCACACAGACACTGGCTGTGTATCAATAGGCACACACACTCATACATGATGCTCAGGTTGTCCACATGCATACAGTGGCACAAATACAGCACACACCCGCACGTATGTGTGCACACAGATACACACATACATGCACACAGAGTAAAGCCTCATGGAAACAGAGACTGTTAGCTCTCCTCTGGCTAGCCTGGATGGCTTTTGCCAGGGCCTTACCAGCACCTGTACCCAGAGGGATCTGCTGAGCCTCCACCTATGGTCACTGGGAGACAAGAAGGTCCATGTGAGGCAGGCCAATCACTGCTCAGAGCCTCCTCCTCCATGTAAGGGTGTTCCCCTGAGTTCAGTATGACTTGAATAAAGGCCTCTGCCTTACCCACAGTGGTAAAGCCACTACAGGAAGTTCTCTCAATCCTTGTGGCCTGGAGGCAGCCTGATGTCGCCCCACCCCTCCTCCTGTTTTCTCAAGCATCACCTGCTATTTTGGGAGGGTGAAGGACACATTTTCCCGGCTCTCTTTGCTCTCCACAACACCCAGCACAGAACCGTGTACACAGAATATCTTCAGCCACTGGATGCCTGTGAACTAAATTCAGCAAATGGAAACCATAAGTACCTGTTACCAGGCCACCACTCTTACAAAACACAGATTCTTTTTTTTTTAAGATTATTAATTATTATATGTAAGTAGACTGTAGCTGTCTTCAGACACACCAGATGAGGACATCAGATCTCATTATGGATGGTTGTGAGCCACCATGTAACTGCTGGGATTTGAACTCAGGACCTCTGGAAGAACAGTCAGTGCTCTTAACCACTGAGCCATCTCTCCAGCCCCAAAACACAGATCCTTAAACATAAGCAACCCCTTCATTGAAATGACTGTTCAGTTATCATAGGCAAGGACAAGGCCATAGCTCAGCAGTAGAGACCTTGAGTGAGGCATAGGTTTGATCTCCAGCATGACAAAAAGGAACACACACACACACACACACACACACACACACACACGCATACACATACTCACTGGGGGGCATAGGAAACCTCCTATTAATGGCATGTTATTTTTTAAAATTACATTGATAGATGGATGGATGGATGGACAGATGGATGGATGATTGATTGTGTTCATGTGTCATGGTTCATGGGCAGAGCAGAGGACAACTTGCAGAAGTCTGTTCTCTCCTTGCATGTGGGTTCCAGGGAGACTGAACTCAGGTCTTCAGGCATGGTGGCAAACCTTAACCTCCTGATCCATCTTGCAAGCCCATTTGATTTCCTCCTCATGAACTGTAGTGATGGTTTATGGCATGTAATTTTCATTCCTTTCTCTTCCAGGGTCAGAAAGCATGGATAGAGAAGACATTTTGCAAAAGGGAATGCATCTTTGTAATTCCCAGTACAAAAGACCCTAACAGGTACGTTGGATGCTATTGTAGAGACATGATCTGAGTCGGGGCTGGTCTGGCATGTCTCAGCAACTATTCTTCTAATGTCTGCGGGAGAAGGGAGAGGAGGCAAGGACATACTAGACTCTCTCCCCACCAGAGCCATTAACACAGAGTCACTGGGCTAATCAGCTCCTCTTTTGAGAGGTAAATGCCTTGGCAGAGGACTTTTCTTTGCAAGTCTCTTGTGAGCTCTCTCTCTCTCAGCCAGATGTGAGCAAGATCTTTAGACAGTTTCAGGATATCTTCCAGAGCCTCAGCAGTCCAATCCTCTAATTTATTTATACAAAAAGGAACTTCTATCCTTGGGAAAGATGTGAGTGGCTGAGTGAAATGACTATGGCCCTTGTTCACAAGATGGCCAGCACTTTCACTTACCCGTCTGAAATGCACGCTATAGGGTAAACAGAAAGCCTTCCAGAACACTCCAAGCAATCTGGAGGATTCAGCTGAAACATATCCTCTGAGAGAAAAAGCTGAGGCTAAGCTGAAGGGTAGGGGAGACTTATCCATAGGCTCTCCCTACAATTCATGTGAGCAGGGTCACTGGTGCAAAATGACACAGATGCCTGGGCATCACCTGGTTCAGATTTGCTTGGTGTCTCATGTCCTCAAGCTCCCTGTTCCCCCCACCTCGTACCCTGCAGGCCTTGCTCCTCACCTGAACCCTTGTATAACTAGGAGAGAAAGAGGCCCTCCATCACAGGCCTTCTCTTTCTTCATCCCAGCAACTGAGAGGTGAATAGCTCTGTCCCTCCTCCTCCTGCTATGATGGACAAAGGTCTTTGAAACAATGAGAAAAAGCTTTCCTCCTTATTCCCCAAGTCACATGACTTTGTTATAGAAAATAGAGAGAATCAAGTCCTAAAATCCACATGAACTTTTGTAAGAAGGGAGGGAAGGAGAGAAAAGAGGAGCAATGCTTTGGTAAATATTATTTTGAGGGTTTTACCCCACTTAGACCATATAGTTTCTGAAATAAAAGACGCAGAAGCCTTAATATTTATAATAAACTTAAAAGCAATAGAGCTGGGCAGATATCAGCCCTCCGTGCTATTGTGTCTACTTCCTTGTCAATAACCCAAGATATCACTTGCCATGTTTCACCTAGACTACTCCTACTTCATCGCTCATGATATACCTAGCCGATAGCAACTTCTCTCTCTTTCCTCTTATTCTTCTCCTGCTCCCTGCCTCGGACCCCAAGCTGGGGAACCCTCATTCTGTCCTCTCTCTTCTGCCCAGCCCAGACTTTAGGCATCTTAGTAACCGATCAGGAATAACTTGGGGTGGGGGGTGGGGGGTGGGGGGAAGGTTCACACAACAAAAGTTGGTGTCCGTGAAGATTTCGGGTAGAAAAAAGGTTTCACTGAAACAAACAAGCAAACAAAAAAC***CTTGAAGAACCATGCGGCACCCTAGAATTCCAGAAGGTCACTTGTCTGTTCCAAGGCTATGACTATACTTGCTACAAATTGTTCAGCAATGTTCACAGGGCCCTGTTCATGTTCTTGAGGCAGTAGAAAGATAACCAGTCATCCAGTGAGAAAGAACAGCCATGCAAAGTGTTGCAAGCAGACTGGTATATAAGTAGTGATGTGTTGCTCTTTGAGAAATGGGCATTTCAGGGGACACCTCTGTCCCTGATAAAGCTGATAACTAAGATATTCCTGGGGGCCCTGGCTGGTGCCAGCTTGTCCGCTTAGGCTACCTTACACTCACTGGTTGGATGGAAGGAAGGAAATGCTGCTGAATGGCCTGCTTTGCTTTGCTTTCAGATGTTGCTGTGGTCAGCTCACTAACCAGCACATCCCCCCTCTGCCGAGTGGGGCTCCCAGCACAACAGGAGAGGACACCAAGCAGGCAGACACGCAGTCCGGGAAATGGTCTGTCAGCAAACACACCCAGAGCTACCCAACAGACTCCTATGGGATTCTTGAATTCCAGGGTGGGGGTTACTCCAATAAAGCCATGGTAAGAGGGGTGATGGGGTGCTGTACAAAGCAACTTAGCCTTCACTAGGGCCTGTCTCCTGGCCCTTAGGATGCAGAGACCTAGGTCTTCTTCTGGCTTACAGCTCAGCTTTCATAGGCACATTACTGCAAGTCCATGAGATGGTTCTGTGCAGATGGATTTTACCCATTCATCAGAACTTGACATCACTGTACATGCAGCCTTGGAGAATCTACCCCAAGCTCTGAACCTAATGTACAAAGCACGCCCAAGCCTACCCTTGCACACATGCGCCTTTGCCCCATCCCCAGTGTCCTCCTTTGCTTTGTATTGCTATGATAGATACTGCGACCAAAGAAACTTAAGAAGAAAAAGGGTTTATTCCATCTTATGTTTCTGGGTGATATAATAGGTCATCTCTGAGGGAACTCAAGACATGAACCTGGAGGCAGGAACTGAAGCAGAGGCCACAGAGGAGTGCTGCTTACTGGCTTGCTCTCCATCACTTTCTCAGCCTGGTTTTTTTAATACAGCCCAGGATCACCTGCTCAGGGGTGGTACCCTCCACACTGAGCTGGGCCCTCCCACATCAATTACTAATCAAGAAAAATGCCCTACACACTTGCCCACAGGCCAGCCTGATAGATAAAAAGCATTTTTACTCTCTCTCTCTTTTTAAAGAATTATTTATTTTTTTATGTATATGAGTACACTGTAGCTGTCTTCAGACACACCAGAAGAGGGCATTAGATTCCATTATAGATGGTTGTGAGCCACCATGTGGTTGCAGGGAATTGAACTCAGGACCTCTAGAAGAGCAGTTAGTCTCTTAACCACTGAGCCATCTCTCCAGCCCAAAGTATTTTCTCAATTGAGGTTTCCTCTTCCCAGATGACCCTAGCTTGTGTCAAGTTGGCAAACAAACAAACCAATCAACCAGCATACCTACTGGTGAGAAAGTTGTTTAAAACAATATTAATAACCAAGGGCTCTCCATCCTTCTCAGTCCATGTGGAGTCTCTGCAAGCATTTTCCTCTGAAGTCAGCTTCCCTGTGTCTTCCCTTCTCAATAAGAGGAAGGAGGCAGTCTCTCATAGTTTGCCATAGGTAGGGCTGCTACCTATATTTCATTGGTCTAACATTATGATTCAAATACAATTGTTATGTTCTGAATACCACAATATAAGGTTCTCTGGGATGGCTTCCTGTATATTAGCATGGGCACACATAGACAGCCCAGAGGGGCCATGGCCTCTGGACACTAATGTCTTTTTCACCCTGAAGAGAAGAAGCACCACTTTCATGACCATGACCACTTTCTTCCTCACTCAGCGATAGCATTGCTCAACATTCCAGACCTTTGTTCTAGCCGAGTCAGTTTGGAAATGATTCTTTCAAGGCCAAGTTTGCAAATAAGTCACAAGGCACTCAGGTATGTTTGAATGTCAGACAAAAGAAGAGACCATTTTTAGTACAACTCTATACCAACTCTTATGTGATGTCCTGCATTTAGTAAAGATACTATGCTAAACCATTTTAGATATTCATCTAAAATTCAAATTTAGACAGGCACATTAGCCTGGTCAGTTGACACTACCATACTTTACATGAGGTGATTTATAAACAGAAATATACTCTATGCAATTCTCAAAGCTAAGAAGCCTCCAATCAAGGTCTCAATTGAGTATAGTTTCTCCCCTTGCTTAATAAGTGTTACCTTCTTTTTTTTTTTTTTTTTTTTTTTTTTTTTTGGTTTTTCGAGACAGGGTTTCTCTGTATAGCCCTGGCTGTCCTGGAGCTCACTTTGTAGACCAGGCTGGCCTCGAACTCAGAAATCCGCCTGCCTCTGCCTCCCGAGTGCTGGGATTAAAGGCGTGCACCACCACGCCCGGCTAAGTGTTACCTTCTTGGTGTGTGCTCACATATGTGAGAAATGAGCAAACCCCTGTGGGCCTCAGAAGCACCATCTCTTATCTACCCTTTACCCCTGCTGCACTAGGATGGAGTTTACACATGTTCCTGTCAGGCTGACACAATCCCCATCTCCAGTTATGGTCATGTTAGGGGTTGGTGCTTCAACAAGGAATTTGGGAGATGGGTAGAGTACTGATCTTCAAATAACACTGCACAATAAGATAACATTTGATAAAAAAAAAAAAGAACAAATTAATGATATAAACCTATATGGTTTGATCTCATAAGCATTATTCTAAGTAAGAGAAGCCAAAAACCGTGTACCTTGTGACTCAACTTAGACAGTATTACTAAAGGTAAAACTCTGGAAACAGAAGCCAGTCAGTGATGCCAGGAATTATGTGTTCAGCAAACACTTGAATCATGGTCCTGTATTTTCCTTTGCTGAGTCGCCCTTCTTTCCCCAGGTTACCTTTAGTCAGTTGGCTGCAGATTAATGCAGAGGGCCCCAAGATATAGAGAGCCTCCAGACACTGGGGGAGTTCTTGTTTGGACACGTGTCATATCCCTGTTCCCCTTTGACAGGGACACGAAGCCAAGCGCGGGCGCGCTGAGCACCACCCCTTCTTTTGCAGTACATCCGAGTCTCCTACGACACCAAGCCAGATTCCCTGCTCCACCTCATGGTGAAGGACTGGCAGCTGGAGCTCCCGAAGCTCTTGATATCTGTGCACGGAGGCCTCCAAAGCTTCGAGATGCAGCCCAAACTGAAGCAGGTGTTTGGGAAAGGTCTGATCAAGGCTGCCATGACCACGGGGGCGTGGATCTTCACCGGGGGTGTGAGCACTGGTAAGAGCGGCCCCGCCCTTGCTGTGAGTCTGCGCTGATGGACTCACTGAAGCTGCAGAGGGCCTCCCAGTAAGAACTGGCTGGAAGACTCGACACAAGGAAGCCTGATCCCAACTGTAACAGCATGTTGAGCTTTACTCAACATGGTTTTTACTATTATCGTGGTTGATTTGTTTAGATAGTACAAACATACTTTTGTTAGGTGGCTTTCCATCACCATGACTGACAGCTGAGATGATCAGTTTATAAAGGGAAAAGGTTTACTCTGGTTCACAGTGTTAGAGGTTTCAGCCTATGGTCAGTTGGACCATTGCTCTGGGCCTGTACATATCTCATGGTGGGAGTACACAGCTCTTCAAGGGCACTTCCCAGTGACTGGCAGACCACAGGCTGGGTCCTTTCTCTTACAGTGTCCACCACTTCCCAATGGTGCCCTGCTAGACACCAAGATCAAACATGGGACTTTGCAGGGACAATCGAGATCTAAACCTCAGTGAGCATAACAGCGATGATAGTGAACCTCTGAGTCAGCTAGTCTGTGCCATTCCCTGAGAATGAACTGAACCATATATGAATTAAGGAACATCCCTGGTGGCCTGGCTCTTACTGATAGCAGAATGTAGCTAGGACTACGAGTGAGACCCAGTGACTTCCAGCTTTGGGTCATAGAAATCCATGCATAGCCGTTTGGGGCTTTGCTCATATTTTCCCTACTCCAGGAGTTAACACAAGATCAATGGTTGTGATGAGCACAGACTTGTGGGATGTATTCTCTTTATGAAGGTTCCATCCCTGATCCACAGGGATTATGGAGGCTCAGAGTAGGCACCCCTGAAGTTCATCAGTGTTGATGTGGCATGATGTGGCTCAGGAGGTCCTGAAATGTGGCATGTGGCTGCTCACAGGTGTCGTCAGCCATGTGGGGGATGCCTTGAAAGACCACTCCTCCAAGTCCAGAGGCCGGCTCTGTGCTATAGGAATTGCTCCCTGGGGCATGGTGGAGAACAAGGAAGACCTGATTGGAAAAGATGTAAGTTTTCTCTGAAGGCACTGACAGGTTTTAAAAAATTGAAGTCTGTATTTTGAGACTGTGGTAAATTTGTATATTGTTATAAGAAATACCAGAGAACACCTTTCACTCATTCCTCTGGTGGAAGGTTCTTGAAAAATGGTAGTATAATCAGGATGCTGACATTGCTGCCATCAAGATACCTCATCAACTGAAGGAAATCTCCTGTTGTCCTTTAATATCTATACCAATCTCCTCCCATCTCTCCTTCCTCCTTAATCCTAACAACCACTAAGGTGTTACATATCTCTATCATTGTTGATTATGTTCACAATGTGGTATCACATAGTATGCAACCTTAGGAGGATGGGTTTTTCCCATATGGACACTCAGATTGCTATGGCTTCCTTCTGTGATATGGATAGACCACTGCAGGTTGTTTAACCACTATCTCTTGAAGAACATCTAGACTGTTTATCTCTGGTCTATTAAAAATAAAGTTTCTATAAAATTTGTGCTTAGGGTGCTGGAGAGACAGCTTAGTGAATGGGTAAAGTGCTTGCTGGACAGGCATAAGGGCTTGTGTTTGTATTCACAATACTCACATATAAATCCAGGTGTAGCGTGTGATTGTAACCTCAGGGCTGGAAATGAAGGCAGGAGGTTCTGGGGCTCATTGTCAGCCACTCTAGCAAGCCAGTGGACTTCATGTTTAGTGAGGGACCTTGTCTTAAAAATAACCTCTGATCTCCACTGGCATGTGTAGCTACCACAGATGCACACACAGAGACACTCACCCGTGCTCAGGATTTTATGTTACTGTTATTTTTCATTTCTCTGCAGTAAATACATGTTCAATTATTGGGCTGCCACTGTCTAAAGCAAACAGTTTTTTTCTTTTTTAATACATTAATTGAGAAAATAGCCTTCAGTGTGTAGGGTAGAGAAATGACACTTAAGTTTACATGTGGTAGCTGAGCAGTGGTGGCACACACCTTTAGTTCCAACATTCATGACAGGCAGAGGCAGAGGCAGAGGCAGAGGCAGAGGCAGAGGCAGAGGCAGAGGCAGAGGCAGAGGCAGAGGCAGAGGCAGAGGCAGAGGCAGAGAGGCAGAGGCAGAGGCAAGCAGATCTTTGTGAGTTTGAAGCTAGCCTGGTCCCACAGTCTATACAGAGGCCTACGCAGAGAAACCCTGTATCAAAAAACAAAACCAAGTTTATATGTTGTGTGTTTACACTCATTTCTTGTGTCTCCTATGACACTTAATATCTAAATTCTGCAGCTTTGATTCACATCATGCTTTCTGTCAGGTAACAAGAGTCTATCAGACCATGTCCAACCCTCTGAGCAAGCTCTCTGTGCTCAACAATTCCCACACTCACTTCATCTTGGCTGACAACGGCACCCTGGGCAAGTATGGTGCTGAGGTGAAGCTTCGAAGACAGCTGGAAAAACACATCTCCCTGCAGAAGATCAACACAAGTAAGTCCTGGGAGGCCATGGGCACCAGCTCGGGACTGACTCTACCAGGGTGGAAACCAGCTGAGAAGGAAATTACCAGAGGGTGCTGATTCTTGCTAGGTCCATGGACTATGCTCAAACTCTTTGCCCTAGCCACCTACCAAATCACCCTCCCAGAGCAGTCATGGGATGTTTTGCCCAGGTTCACAATTTGGTCTGAGACTTACCCTTGCACTATTTGTCATGGTTGTCATGGTGATGGCACTGCCCACCATGTTGCCCTCGGTCTTGTTCAGTGTACTTACTGCTATTATTTTCCTTCATGTTTTATCAGGAAAAAAAAACCTAAAAACTTGTTCCCAAAGATTTGGTATAATTCAGATTGTGACCCAATGAGTGTTGTAGGATCATTACATATCTCTATAGCTCTCAGTCTCCCACATGGACTAAGATGGTGAGATAAGGAAATCCTTGGACTTGGCTACAGGGACATCTGTTCCTGATGGTCACCATACTTGGTGGTCCAGATGGCACAGCTCACTGCTGGGGGGAAATGTGAGGATAAATTAGAGAGAAAGGACTGGCTCCCAACCAGATGAGAGGACCCATAACCTTCTGGGAAATGCCCACATGCAAAACTTCTGCCAGACAATTATGAAATGATCTCACAGAATATTTCATTCTTATCATCACCAGGAATATCTTTGTGCACCACAATCTAGAACTAGGAAAATGTCAGAATGTAAGGAAGTGTGGTATCACTCAAGTGGTTAGAACTGGGGTGC***GCTAGCGTTAACCAATTGTATCACGCGTATAACTTCGTATAGCATACATTATACGAAGTTATGCCACTAGAGGATCCCCGCTACTCCTAAAGGCTATAGATTTCCAAGAATCAGCTTAAACTATTTCTATTAATTCTCTGAGAGTTTTATACAATTTAGTTTTATACAATTATTCTATTCCTCCCCTACTTGTGCCAGATCTACCCCCACCTCCCAAACAGCCCCTGAATTCATGTCCTCTTTTAAAGAAATGACTACTTGACTCCTACTGTTCTGACCATGCAATCACAGGTGTGGGGGCATCCACTAGAGTGTGGTCAACCTATCAGGGCCGCACACTTAAAAAAAAAAACTGACTCCCTTCCCACAGAAGGGAATTTGTCAACTCCTCCCCACCCTCATGCTGGAATACTGACCGGCTTGATCCTGTGCAGCCAGCCACACTGTGAGTTCATGAGTGCGGAGGTCCTAAGAATCAGATCTTGGTGGATAAATCAGTTTGATTTAGTGTTTTTGGACTTGTAAATT**CTGCTTGGACCTGTGACCTGTGGGCTTCCCTTTGTCATCCTTTGCCTAGGCCTCTGAGTGAGGCAAGGACAGCAAAGGGGGGCTCAGTGGTCACCTCTACTGCAGA**GAGTTCAGAAGCCTAGCCTGAGCCAAGAGCAAGTTCTTCTCTGCTTCTGGAAATGAAGTCGCCATGATCCTGACGATGTAAAAATCCCAAGCACGCTTGGATGGAAATCTCAGAGACAGACGATGCCACCCTGATCCATTTTCCACTATTTCTTTTCTCTCTCTCTCTCTCTCTCTCTCTCTCTCTCTCTCTCTCTCCCTCCCTCCCTCCCTCCCTCCCTCCCTCCCTCCCTCCCTCCCTCCCTCCCTCTCTCTCTCTCTCTCTCTCTCTCTCTCTCTCTCTTTCTTTCTTTCTTTCTTTCTTTCTTTCTTTCTTTTTTGAGACAGGGTTTCTTTGTATAGCCCTGTTTGTCTTGGAACTCACTCCGTAGACCAGGCTGGCCTCGAATTCAGAAATCTGCCTGACTCTACCTTCTAAGTGCTAGGATTAAGGGTGTGCGCCACCACCGCCCGGCTTCATTTTCCACTATTTCTTATTCATGGCTTAGCCCAGTGGGTCCCACCCTTCCTAATACTCTGACCCTTTAATACAGTCACTTGTGTTGTGGTGACCCCTCAAACCATAAAATTATTTTTGTTGCTACTTCATTACTGCAACATTGTTACTCTTATGAATCATAATGTAAATATCTGATATGCAGGATATTTGATACGTGACTCCCCTAAGGGGTAGAGACCCACAGGTTGAGAACTGCTGGCTTAGCCTATGCCATATCTTCACCATTTGCTGGCTGGAATAAACTCAACTGTAATTCTTCGTGGGATTGTGTCCGTGTCGCGAAGTTCCTATACTTTCTAGAGAATAGGAACTTCGTTCGAACATAACTTCGTATAGCATACATTATACGAAGTTATGGTACCGACTCGAATTCATCGTACTGGCCATCTAACAGGAGACAACAAAGTTATCCTGAATCCTTAAGTATCCACTGGGGAAAACAAAGTAGAAAAAGAGAGACTCTGAATTCACTCATTGATAGTCTCAAGGCTTCTTATAGCCCGTGCTGGCAGATTTTATCGGAAGCTCTTGAGAACCATAACATCAAGGACATTTCCTTCCTCTTGTCTCTTGTAATGTTGCTCACAACTACAGAAACCAAGCAAAACCTGATCAGCAGTCAGTGAGCCTGGGTGTTGCCAAGGTCCAAAGCCCAAAACTTCACTGGTCACAGACAGAAAAACATTCAGGAGAGAAATTCTGTCTTTAATTAATGTGTATGGAGTTTTTTCCCAGTTGCTATCCCTCAGAAACACAATATAATAACTACTTTCATGTCATTGCATCATGCCAGGCGTTATGAGTCCCCTGGAGATGGCTTGAGGCACACAGGAGAAGATACATGCAGCTGCTACATCATTTTATATAAGAGTCCTGAGCATCTTGAATTTGGTGTCCAGGGGGATGTTATAAGGTTCTGGAGCCAATCCCTAGTAGATGCCTAGGAACAACTCTATAATATGTTCTGGCCAGGCAGAGATGACATTTTGAAGTAGGCAAGTTTGTCACGAAGCCTCCTCTCCAGCACTTTCCCCCCAGTATTATCTCAGCTTCCAGAATTCTCCCATCACTTCCTGCTGGATCTATCACCAAAAATTTGTCTATTCCTACTGTGCCCTTGAAGAATTATATTATTTATTTATTTTAAATCAGAGTAGACTCTGGAAATAAGCCATTGGCAATGGGCTAATAGTACTCCACGCTTTTACCCCTAAGGTAATATCCTGGGGCCAGAAGTGTTTTAGTCTTGGATTTATAAATTTTGGACATATTTGCATACCCTTTACCAATGATCATCTCCAATCCAAATATTTGGTCTTGGAAGATGTCTTGGGGACGGGTGATGCTCAACCTTTTCTCCCGTATTCGAGGCTCTGGAGGCAGCAACAAGAGCAGACTACCACACGAGTGCCTCTTTGACTTCCCAAACACTACGTAAAAGCCAAATCTATTCTTATATTTCAGGTCTGTTGCTCTTCTTGAGATAAGTGAGGTCCTGATCCTTTTACAACTGGTCACCTGAGAGATGGCGCTTTAATGAATGGTCCAGCAGTGCTTTACTGATAGAGGAGAGGCCCTAGATTGGGCTGAATTCCCCTCTGGCTCAGTCCAGCCCTCCTTATACTGGATCATTGTTGGAAGACAGCCCCTAGCATTTCACACCTAGGCAGGCAGAAAAGGAGCCTTCATAGCAATGCTTTTAAAACTGGCCGGGTGTCCCTGTGTTGGATTCTTTACCAGAGTTGGGTCACATGGGCGTGTCAGGGAGTTTAAGAGTTCTGAAATTTGGAGTTTACAGTGGGGGGCCATGATATGAAGTTCGAATGCAAGAATGAAGACAGTCACCCTTCGCTTGACTTAGTCGAAGCATTGGTCAACATTAGCACTACAAGGAAAGCCCTGTGGCAGGAAGTTTGTGCTTTTTGCTGTTTCGGCATTTAATGCAAGTTGGAGCTGTTCATGTTTGTGTGAGCAACAAGTGTGGTTGTGTTGCAGGGCTGGGCCAGGGTGTACCTGTCGTGGGCCTAGTGGTAGAAGGTGGTCCTAACGTGGTTTCTATCGTCCTGGAGTATCTCAAAGAAGACCCTCCTGTCCCTGTGGTGGTTTGCGATGGCAGTGGACGTGCCTCTGACATTTTGTCCTTCGCACACAAATACTGCGACGAAGGAGGGTAGGTTTGGTGTGTGACAAGGGTGAGCTCTTAATGGCTTTTTGTCAGACTGGGGAAAACATCTGACACCCCTCAGATATCCCCCAAAATGTCTAATTTACAAGGGGTTCTCTTGATCTTCTCTCCTGAGCTCCCGAGATCAGCATGAGAATGCCCAGGAGCTCCTGTTCCTGACTGAATCCTCTCTGCCGACATCTCTCTTACAACTTTATTTTACAGTATTAAGTCTTGCAAAGAGCTAAACGCAGGCCAATTTTTAATGCTGCTTTTAGACTGGGTTGGCTTTTTGTCTCCTGTACACAAATCTCATCCATTAGCAGTCCAAAATCAAAAGTACTGGGTGGGCGAAACTGTCTGGCTCACTTGGGAGCTGATCTAATTCCCTTGCTTCCTTCCCTCTCTTTACCCCTTTGAACAGAGTCATAAACGAGTCCCTGCGGGACCAGCTTCTAGTTACCATTCAGAAAACATTTAATTACAGCAAGTCCCAGTCGTATCAGCTGTTTGCAATTATCATGGAGTGCATGAAGAAGAAAGAACTCGTGAGTGTCTTGAATTGCATTACCAAACAAGTTCCAAGGAGAGAGATCTTTCTGTGTCTAGCTCTGCCATGCTTGCTTGATGGGCCATCTGCATACTCAGTTGACCTTCTCAAAAGGCAGCTTATGAACTCATCCCAAACTAGCTGCCAGTGCAACCCTGAAGGAATGGGAAAAGCCACAGAGCAGTGGTTCTCAACCTGTGTGTCGGGACCTCTTTGGGTGCCAACCTTTCACAACCTTTCACAGGGGTTGCTTAAGACCACTGGAGTAACAATAAAAATAATTTTATGGTTGGGGATCACCACAACATGAGGAATCGTATTAAAGGGCCGAAGCATTAGGAAGGTTGAGAGCCATTGCCACAGAGGACCCAGGTGTATCCAGACCTTAGTGAACATAAGGTGATGCCAATCTTGTCACTCAACTCAGAAATTCAAATCACAAATCAGATGTAAAGTCCCCAGGGAAGAGAGACACAAACATGTCATTAGATGTGATGGGTCACAAAGTCAAATTCACTTCCCTTTGCAAATGCAGGGCTCATTTCTCATTTCTTCCTTCAGCTTCCTTCAACCTTATAAGGCGTAGGCACACCTAGTTCATGAATGATGACATATTCAAAATTCTAAATTCATGTTCACATATTCCTGCTGATTAGGCATGTGTTCACAAGCTCAGATAACTATTTAAATATGGAGCTATCCTGAGGTGCTTACCCTCTTCCCAAGGCAGGATGATAGATTCCAATCCCGGGCAAGAATTCCATCAGCAAGAACAACATGGAAGCTCCTGGGGACTGAAAACTGTGAACATCCATCCAACC
